# Serinol-Based Versatile Disulfide-Reducing Reagent †

**DOI:** 10.3390/molecules28145489

**Published:** 2023-07-18

**Authors:** Babita Kushwaha, Sinenhlanhla N. Mthembu, Anamika Sharma, Fernando Albericio, Beatriz G. de la Torre

**Affiliations:** 1 KwaZulu-Natal Research Innovation and Sequencing Platform (KRISP), School of Laboratory Medicine and Medical Sciences, College of Health Sciences, University of KwaZulu-Natal, Durban 4041, South Africa; babitakushwaha46@gmail.com (B.K.); sneniamthembu@gmail.com (S.N.M.); anamika.aug14@gmail.com (A.S.); 2 Peptide Science Laboratory, School of Chemistry and Physics, University of KwaZulu-Natal, Durban 4000, South Africa; 3 Department of Natural Products and Medicinal Chemistry, CSIR-Indian Institute of Chemical Technology, Hyderabad 500007, India; 4 CIBER-BBN, Networking Centre on Bioengineering, Biomaterials and Nanomedicine, Department of Organic Chemistry, University of Barcelona, 08028 Barcelona, Spain

**Keywords:** cysteine, disulfide-reducing agents, monothiols, dithiols, phosphines

## Abstract

Here, we report the synthesis of disulfide-reducing agents 2-(dibenzylamino) propane-1,3-dithiol (DPDT) and 2-(dibenzylamino)-2-methylpropane-1,3-dithiol (DMPDT) from serinol and methyl serinol, respectively. DPDT was found to show greater stability than DMPDT. Hence, the effectiveness of DPDT as a reducing agent was evaluated in both liquid and solid phases. The reducing capacity of this agent was comparable to that of DTT.

## 1. Introduction

Free thiols and disulfide linkages have unique chemical and physical properties and they are key players in many biological processes [1,2]. Although cysteine (Cys) is the least abundant amino acid, it is an integral part of the functional sites of proteins [3]. Thiol present in Cys (from two residues) can form disulfide bonds by undergoing oxidation, a process that renders a tertiary structure to the protein and confers high stability to the final molecule [2]. Cys residues are located mainly in functionally or structurally important areas of proteins, where they act as stabilizing, catalytic, metal-binding, and/or redox-regulatory entities [4,5,6,7]. Furthermore, disulfide links in synthetic peptides enhance pharmacological characteristics by increasing rigidity and hence, stability [8].

**Figure 1:**
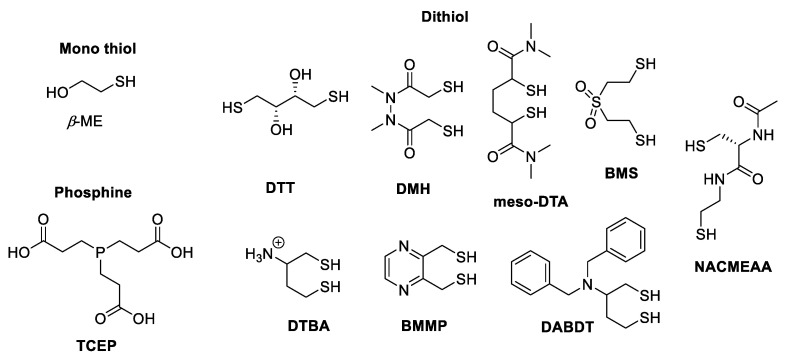
Common disulfide bridge reducers.

Given the significance of Cys and disulfide bridges, disulfide-reducing agents are frequently used to stabilize free sulfhydryl groups and reduce disulfide bonds in peptides and proteins [1,9]. The most ubiquitous dithiol reducers are broadly classified into two categories, namely thiol-based reversible reducers (monothiols and dithiols) and phosphine-based irreversible reducers (Figure 1) [1].

Each category has advantages and disadvantages. Thiol-based reducers are either monothiols or dithiols. The former example, *β*-mercaptoethanol (*β*-ME), is usually foul smelling and is required in large amounts to prevent the formation of mixed disulfides [10]. This issue is addressed using dithiols as they form stable six-membered cyclic by-products. Moreover, dithiol-based reducers are non-malodorous and are kinetically faster than monothiols. Among dithiol-based reducers, dithiothreitol (DTT), although expensive, is still a powerful reducing agent and has long been the reagent of choice for the quantitative reduction in disulfide bonds [11]. However, DTT is non-functional at pH < 7 and it also has a very short half-life (*t*_1/2_) in solution. Thus, a fresh solution is required before use. In addition, *N*,*N*’-dimethyl-*N*,*N*’-bis(mercaptoacetyl)hydrazine (DMH) [12], meso-2,5-dimercapto-*N*,*N*,*N*’,*N*’-tetramethyladipamide (meso-DTA) [10], and bis(2-mercaptoethyl) sulfone (BMS) [13] were developed to address the limitations of DTT. These reagents were then followed by the development of (2*S*)-2-amino-1,4-dimercaptobutane (DTBA) [14], 2,3-bis(mercaptomethyl)pyrazine (BMMP) [15], 2-(dibenzylamino)butane-1,4-dithiol (DABDT) [16], and (2R)-2-(acetylamino)-3-mercapto-*N*-(2-mercaptoethyl) propanamide (*N*-acetylcysteine mercaptoethylamine amide (NACMEAA) [17]. DTBA is synthesized from a low-cost starting material but requires the use of hazardous and expensive conditions like the Mitsunobu reaction [14]. The elimination of the triphenylphosphine by-product is a tedious operation and the presence of the amino group affects the reducing efficiency of certain disulfide bonds due to unfavorable coulombic interactions [14]. Furthermore, DTBA is soluble only in aqueous solutions, thereby limiting its applicability in non-aqueous conditions. To address this issue, we developed DABDT, which is soluble in a wide range of non-aqueous media [16]. DABDT shows a higher stability to air oxidation than DTT in solution, and a good reducing capacity in both solution and solid phase [16].

Among phosphine-based reducers, TCEP works more efficiently over a broad pH range (5.0–9.0) than the abovementioned reagents and it is stable to air oxidation [18]. Unlike the other reducers, the process is irreversible due to the formation of stable phosphine oxide. As TCEP is only aqueous soluble, its usage is limited [18].

**Figure 2:**
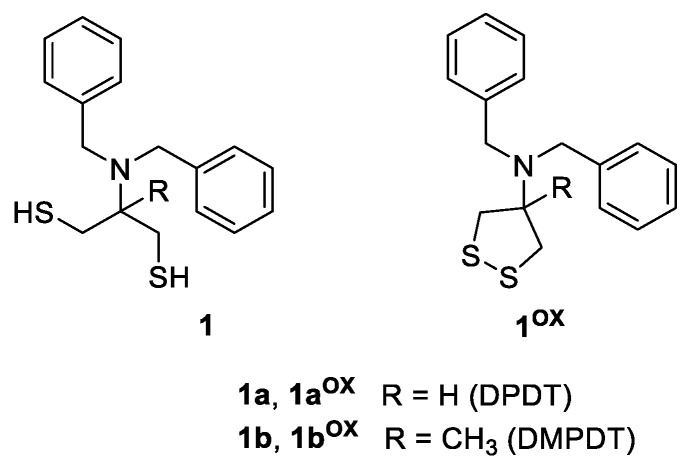
Structures of the disulfide-reducing agents and their oxidized counterparts.

An ideal reducing agent should be non-malodorous and inexpensive. Moreover, it should have a low p*K*_a_ and a wide range of solubility. As part of our ongoing interest in developing cost effective and non-malodorous new disulfide-reducing reagents, here, we developed 2-(dibenzylamino)propane-1,3-dithiol (DPDT) and 2-(dibenzylamino)-2-methylpropane-1,3-dithiol (DMPDT) disulfide-reducing agents to be compatible with non-aqueous conditions (Figure 2).

## 2. Results and Discussion

**Scheme 1:**
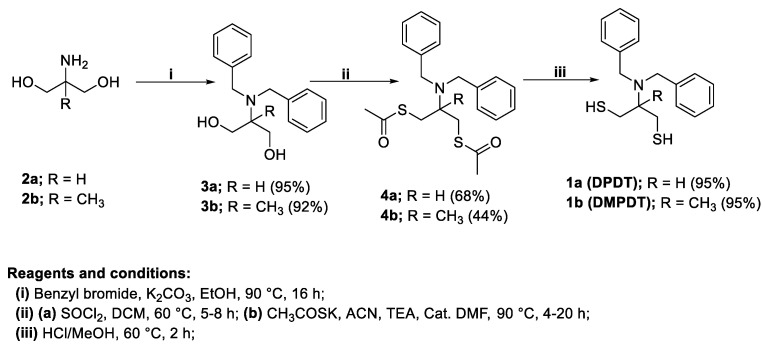
Synthetic route to prepare DPDT (**1a**) and DMPDT (**1b**).

The design and efficiency of novel disulfide-reducing reagents based on dithiol molecules often depend on their capacity to form a stable cyclic compound in its oxidized form [19]. Synthetic dithiol-reducing agents mostly form a 6-member ring (or higher) when in an oxidized form (Figure 1). However, the formation of a 5-member ring, as is the case of dihydrolipoic acid, is also a favored structure [20]. In previous studies, DTBA [14] and DABDT [16] were synthesized from aspartic acid to obtain the corresponding dithiol-reducing agents. In the study presented herein, we pursued a starting material that does not contain carboxylic acids, thereby avoiding the use of a stronger reducing agent like lithium aluminum hydride (LAH). Further, the resulting reducing agent forming a 5-membered ring in its oxidized form is used for comparison to determine if it serves as a better disulfide-reducing agent. In this regard, we identified two suitable candidates as starting materials, namely serinol (**2a**; 2-aminopropane-1,3-diol), which would lead to DPDT (**1a**), and methyl serinol (**2b**; 2-amino-2-methylpropane-1,3-diol) for the synthesis of DMPDT (**1b**) following the synthetic route depicted in Scheme 1.

The global synthesis consisted of three steps. In the first, the amino group was subjected to benzyl protection using benzyl bromide. This reaction was conducted in ethanol using a conventional method, affording the desired product 2-(dibenzylamino) propane-1,3-diol (**3**) in excellent yield and with high purity and without the need for column purification (SI Figures S1–S4 for **3a** and Figures S17–S20 for **3b**) [21]. In the second step, the hydroxyl groups were converted to thioesters via an intermediate step, wherein the OH group was replaced with chloride by reacting with thionyl chloride (SOCl_2_) [16]. This was further treated with potassium thioacetate in acetonitrile (ACN) with a few drops of *N,N’* dimethylformamide (DMF) in the presence triethyl amine (TEA) at 60 °C for 4 h to form S, S’-(2-(dibenzylamino) propane-1,3-diyl) diethanethioate (**4a**) in high purity and excellent yield and with no further column purification (SI Figures S5–S8). However, the formation of **4b** took longer than that of **4a,** which could be attributed to the steric hindrance of the bulky methyl group. The reaction was performed at 90 °C for 20 h to afford crude product **4b**. The longer reaction time led to several impurities and **4b** was therefore purified using column chromatography (SI Figures S21–S24). Finally, a reduction in the thioester was attempted using our previously reported method (NaBH_4_/MeOH) [16]. The progress of the reaction was monitored using HPLC. In contrast to our previous report, a mixture of **1**, **4**, monothiol (with one thioester) and **1**^OX^ (in high concentration) was observed. To overcome this limitation, the hydrolysis of **4** was attempted in both basic (aqueous LiOH, NaOH, or KOH [22] in MeOH) and acidic (aqueous HCl in MeOH) [23] conditions. In the case of basic hydrolysis, only **1**^OX^ was obtained as the major product. In contrast, in the acidic hydrolysis, the product was obtained but not with significant conversion. However, when the acidic hydrolysis was carried out under anhydrous conditions (anhy. HCl/MeOH) [14], full conversion was afforded (SI Figures S9–S12). DPDT (**1a**) was obtained with an overall yield of 61%. This yield was greater than that of a previously reported DABDT (48%) [16]. This increased yield is attributed to the elimination of a synthetic step (LAH reduction) and the elimination of column purification during the process. Trace amounts of **1**^OX^ were observed in the final product. However, in the case of DMPDT (**1b**), the product was obtained in a lower yield (38%) (SI Figures S25–S28). For reference, **1a** and **1b** were treated with 6N NaOH to afford **1a^OX^** (SI Figures S13–S16) and **1b^OX^** (SI Figures S29–S32).

First, the stability of the two compounds in solid form was checked after storing the reducing agents at room temperature for 60 days in a vial without sealing and in a non-inert atmosphere. DPDT showed approximately 5% of the oxidized form, comparable to that observed when the compound was synthesized (SI Figure S33). In contrast, DMPDT showed a slightly greater percentage of the oxidized form than the initial compound (SI Figure S34). These observations indicate that DPDT is more stable than DMPDT as a solid.

**Figure 3:**
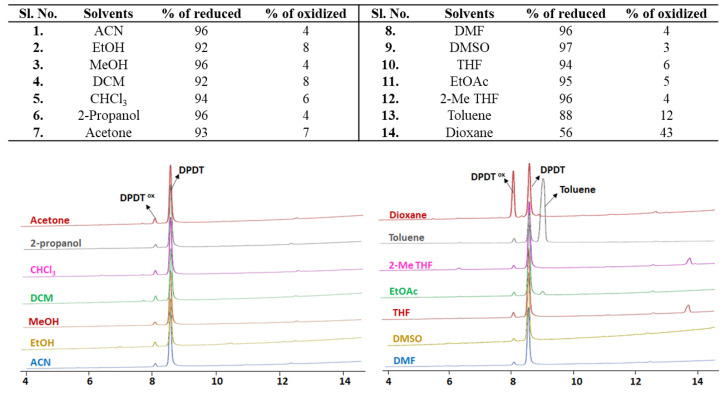
HPLC chromatograms of DPDT in different organic solvents (1 mg dissolved in 10 mL and 1 µL was injected immediately in HPLC (5–95% gradient of ACN (0.1% TFA)/H_2_O (0.1% TFA) over 15 min at a flow rate of 1.0 mL/min and detection at 220 nm).

**Figure 4:**
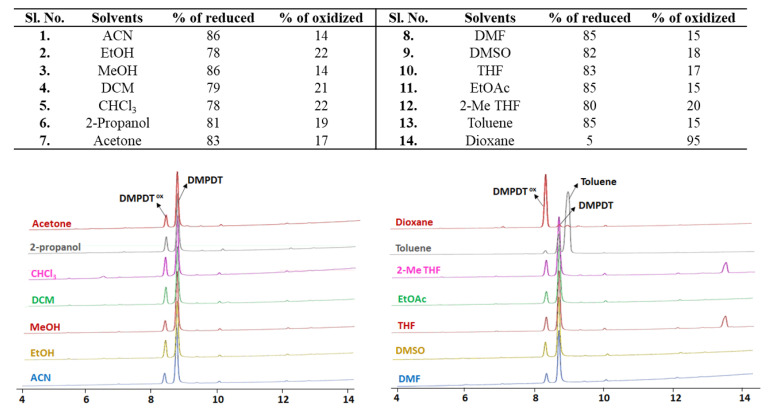
HPLC chromatograms of DMPDT in different organic solvents (1 mg dissolved in 10 mL and 1 µL was injected immediately in HPLC (5–95% gradient of ACN (0.1% TFA)/H_2_O (0.1% TFA) over 15 min at a flow rate of 1.0 mL/min and detection at 220 nm).

Furthermore, DPDT and DMPDT were soluble in a wide range of organic solvents of different polarities (see Figure 3 and Figure 4 for the solvents tested). The solutions were injected into the HPLC to study their compatibility with the different solvents. For DPDT, in all cases except toluene and dioxane, practical oxidation was not detected (the oxidized form was present in approximately 4–8% with respect to the reduced form; Figure 3). On the other hand, for toluene and dioxane, the presence of the oxidized form reached 12% and 43%, respectively. In the case of the latter, this high percentage can be attributed to the presence of peroxides. This result was confirmed when the same experiment was carried out with DMPDT, which showed a 95% formation of the oxidized form in dioxane (Figure 4). In the rest of the solvents tested, DMPDT showed a greater formation of the oxidized form than DPDT. Interestingly, neither compound showed important oxidation in DMSO. However, given the results obtained, DMPDT was abandoned. DPDT also showed full stability in ACN for at least 11 days (SI Figure S33).

**Figure 5:**
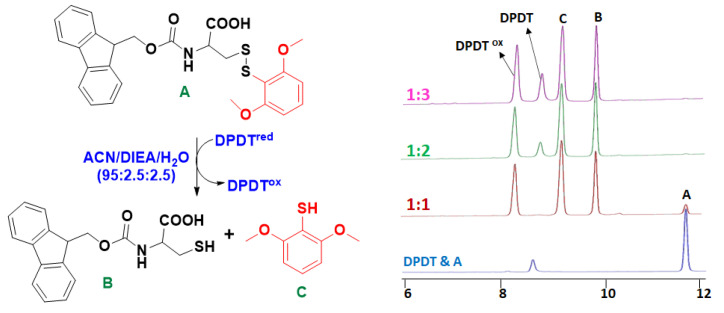
HPLC chromatogram of disulfide bridge reduction in Fmoc-Cys(SDMP)-OH using different ratios of DPDT. (Equimolar solution of DPDT and Fmoc-Cys(SDMP)-OH) were mixed in 1:1; 1:2 and 1:3 ratio with DIEA/H_2_O to obtain final concentration as 95:2.5:2.5, and 2 µL was injected immediately).

Next, we examined the reducing capacity of DPDT in solution. To this end, we used as a model Fmoc-Cys(SDMP)-OH (*N*-a-(9-Fluorenylmethyloxycarbonyl)-S-(2,6-dimethoxythiophenol)-*L*-cysteine), which contains the thiol side-chain of the Cys protected as disulfide. We performed parallel experiments using different ratios of Fmoc-Cys(SDMP)-OH to DPDT, namely 1:1, 1:2, and 1:3 (Figure 5). The reactions were carried out in ACN containing 2.5% of base [*N*,*N’*-diisopropylethylamine (DIEA)] and 2.5% of H_2_O. We previously reported that the addition of H_2_O to the reaction media was necessary to avoid the reversibility of the reaction [16]. As expected, in the case of the 1:1 ratio, the reaction was not complete as some Fmoc-Cys(SDMP)-OH remained in the reaction mixture. This observation is attributed to the fact that DPDT contained approximately 5% of the oxidized form. In the case of the 1:2 ratio, the reduction was complete, and an excess of DPDT was observed. The same scenario was observed for the 1:3 ratio. The best reaction conditions were thus determined to be a 1:2 ratio of Fmoc-Cys(SDMP)-OH to DPDT in ACN/DIEA/H_2_O (95:2.5:2.5). The study conducted using DTT as the reducing agent showed comparable results. The reduction was completed in a 1:2 ratio. However, the peptide dimerized. This process was not detected when DPDT was used, thereby making the latter agent a better choice for this disulfide reduction (SI Figure S35).

One of the main drawbacks of thiol-based reducing agents is that they are easily oxidized in basic medium. However, base is necessary to achieve the thiolate form, which is the active species of the reducing agent. With this in mind, we studied whether the reductive properties of DPDT are maintained in bases weaker than DIEA (pKa = 11.0), such as *N*-methyl morpholine ((NMM), (pKa = 7.4)) and 2,6-lutidine (pKa = 6.7). These weaker bases were tested under the best conditions identified earlier, namely with a 1:2 ratio of Fmoc-Cys(SDMP)-OH to DPDT. The poor conversion of Fmoc-Cys(SDMP)-OH to the reduced form was observed, even when the percentage of base in the solution was increased (SI Figure S36).

**Figure 6:**
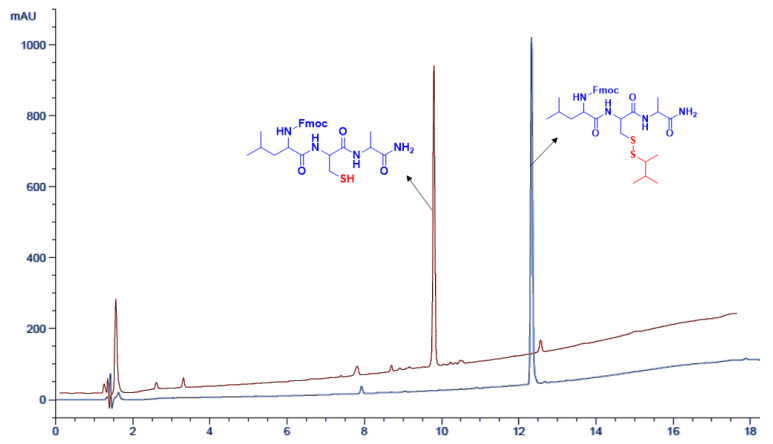
HPLC chromatogram demonstrating DPDT-mediated disulfide bridge reduction in a peptide attached to a solid substrate. (Residue after global deprotection was dissolved in 200 μL of H_2_O/ACN (1:1) and 2 µL injected immediately).

Furthermore, we examined the reducing capacity of DPDT on a protected Cys tripeptide anchored on solid phase. SIT (sec-isoamyl mercaptan) was used as the protecting group of Cys. As this group is more stable than SDMP, its removal is more demanding that in the previous case. The peptidyl resin experiments were designed to use 15 eq. of DPDT divided into three portions. Thus, the peptidyl resin (Fmoc-Ala-Cys(SIT)-Leu-rink amide resin) was subjected to three × 10 min treatments with 5 eq. of DPDT in a mixture of DMF/DIEA/H_2_O (95:2.5:2.5) as solvent. An analysis of the peptides after cleavage from the resin confirmed the almost quantitative disulfide reduction rendering the unprotected Cys tripeptide (Figure 6). In parallel, the same experiment conducted using DTT as the reducing agent (SI Figure S37) resulted in comparable results.

## 3. Materials and Methods

### 3.1. General

All the fine chemicals, reagents, and solvents were purchased from commercial suppliers and were used without further purification unless otherwise stated. DIC and OxymaPure were a generous gift from Luxembourg Bio Technologies (Ness Ziona, Israel). The progress of the reactions was monitored by thin-layer chromatography (TLC) on precoated silica gel plates procured from E. Merck and Co. (Darmstadt, Germany) visualized by a UV lamp (254 nm). Analytical HPLC was performed on an Agilent 1100 system using a Phenomenex C_18_ column (3 μm, 4.6 × 50 mm), and Chemstation software was used for data processing over a 5–95% gradient of ACN (0.1% TFA)/H_2_O (0.1% TFA) over 15 min at a flow rate of 1.0 mL/min and detection at 220 nm. All mass spectrometry data were obtained from a Thermo Fisher Scientific UltiMate 3000 UHPLC-ISQTM EC single quadrupole mass spectrometer in positive ion mode over a 5–95% gradient of ACN (0.1% HCOOH)/H_2_O (0.1% HCOOH) for 15 min. The NMR spectra (^1^H, ^13^C) were recorded using CDCl_3_, DMSO-*d_6_* and MeOD-*d_4_* on a Bruker AVANCE III 400 MHz spectrometer. Chemical shifts were determined relative to internal standard TMS at δ 0.0 ppm (ppm), and the coupling constants were reported in Hertz. The multiplicities of the NMR resonances were abbreviated as s (singlet), d (doublet), dd (doublet of doublet), t (triplet), q (quartet), m (multiplet), and brs (broad singlet).

#### 3.1.1. Synthesis of 2-(dibenzylamino) propane-1,3-diol (**3a**)

Anhydrous K_2_CO_3_ (2.34 g, 17.01 mmol) was added to a stirring solution of 2-aminopropane-1,3-diol (0.5 g, 5.48 mmol) in EtOH (50 mL). Benzyl bromide (2.0 mL, 17.01 mmol) was added dropwise to the above solution with stirring. The resultant mixture was refluxed for 16 h. After completion, the reaction mixture was cooled to room temperature (rt) and volatiles were removed under reduced pressure. The residue was quenched with saturated NaHCO_3_ and extracted with EtOAc (3 × 20 mL). The organic layer was dried over anhydrous MgSO_4_ and filtered. The solvent was removed under reduced pressure to afford crude product, which was then triturated with *n-*hexane and filtered to result in the title compound (**2**) as a white solid (1.41 g, 95%). HPLC *t*_R_ = 5.64 min; ^1^H NMR (400 MHz, DMSO-*d_6_*, 25 °C) *δ* 7.36 (d, *J* = 7.52 Hz, 4H), 7.28 (t, *J* = 7.64 Hz, 4H), 7.19 (t, *J* = 7.17 Hz, 2H), 4.33 (s, 2H), 3.73 (s, 4H), 3.62–3.54 (m, 4H), 2.72–2.67 (m, 1H). ^13^C NMR (100 MHz, DMSO-*d_6_*, 25 °C) *δ* 140.7, 128.3, 128.0, 126.5, 60.5, 59.5, 54.1. LC-MS m/z calculated for C_17_H_21_NO_2_ [M+H]^+^ 272.16, found 272.22.

#### 3.1.2. Synthesis of S,S’-(2-(dibenzylamino)propane-1,3-diyl) diethanethioate (**4a**)

Excess SOCl_2_ was added to a stirring solution of **3a** (2 g, 7.37 mmol) in DCM (20 mL) under nitrogen. The resultant mixture was stirred at 60 °C and monitored by TLC until no starting material was observed. After completion of the reaction (5 h), the solvent was removed to yield a solid residue. A solution of potassium thioacetate (4.20 g, 36.85 mmol) in ACN (with a few drops of DMF) was then added to the residue, followed by TEA (0.1 mL, 0.73 mmol), and the reaction was heated further to 60 °C and monitored by TLC. After completion of the reaction (4 h), the mixture was concentrated under reduced pressure. The residue was diluted with H_2_O and extracted with EtOAc (3 × 30 mL). The organic layer was collected, dried over anhydrous MgSO_4_, and filtered. The solvent was then removed under reduced pressure. The semi-solid residue was kept under vacuum for 2 h, triturated with *n*-hexane, and filtered to result in **4a** as a pale-yellow solid (1.95 g, 68%). HPLC *t*R = 11.70 min; ^1^H NMR (400 MHz, DMSO-*d_6_*, 25 °C) δ 7.36 (d, *J* = 7.12 Hz, 4H), 7.30 (t, *J* = 7.39 Hz, 4H), 7.21 (t, *J* = 6.30 Hz, 2H), 3.64 (s, 4H), 3.30–3.25 (m, 2H), 3.0–2.95 (m, 2H), 2.65–2.61 (m, 1H), 2.28 (s, 6H). ^13^C NMR (100 MHz, DMSO-*d_6_*, 25 °C) *δ* 194.9, 139.3, 128.6, 128.0, 126.8, 57.6, 52.4, 30.4, 28.5. LC-MS m/z calculated for C_21_H_25_NO_2_S_2_ [M+H]^+^ 388.13, found 388.12.

#### 3.1.3. Synthesis of 2-(dibenzylamino)propane-1,3-dithiol (**1a**)

Anhydrous HCl/MeOH (10 mL) was added to **4a** (1 g, 2.58 mmol) under nitrogen. The resultant mixture was heated at 60 °C for 2 h under nitrogen. After completion of the reaction, the mixture was cooled to room temperature and concentrated under reduced pressure. The solid residue obtained was diluted with DCM and washed with H_2_O. The organic layer was collected, dried over anhydrous MgSO_4_, and filtered. The solvent was then removed under reduced pressure to afford DPDT (**1a**) as a white solid (0.74 g, 95%). HPLC *t*_R_ = 8.50 min; ^1^H NMR (400 MHz, DMSO*-d_6_*, 25 °C) *δ* 7.39–7.37 (m, 4H), 7.31 (t, *J* = 7.68 Hz, 4H), 7.24–7.20 (m, 2H), 3.35 (s, 4H), 2.89–2.83 (m, 2H), 2.68–2.66 (m, 1H), 2.64–2.62 (m, 2H), 2.35 (t, *J* = 7.67 Hz, 2H). ^13^C NMR (100 MHz, MeOD- *d_4_*, 25 °C) *δ* 132.0, 131.4, 130.8, 130.6, 67.7, 56.6, 22.9. LC-MS m/z calculated for C_17_H_21_NS_2_ [M+H]^+^ 304.11 found 304.14.

#### 3.1.4. Synthesis of N,N-dibenzyl-1,2-dithiolan-4-amine (**1a**^OX^)

**1a** (0.020 g, 6.59 mmol) was diluted with DCM and washed with 6N NaOH solution. The organic layer was dried over anhydrous MgSO_4_ and filtered. The solvent was then removed under reduced pressure to afford DPDT^OX^ as a white solid. (0.019 g, 96%). HPLC *t*_R_ = 8.02 min; ^1^H NMR (400 MHz, CDCl_3_, 25 °C) *δ* 7.34–7.27 (m, 5H), 7.24–7.15 (m, 5H), 3.64–3.59 (m, 2H), 3.53–3.45 (m, 2H), 3.10–3.09 (m, 1H), 2.97–2.91 (m, 1H), 2.68–2.54 (m, 2H), 1.61–1.50 (m, 1H). ^13^C NMR (100 MHz, CDCl_3_, 25 °C) *δ* 139.2, 139.1, 129.2, 128.5, 128.48, 128.43, 127.34, 127.30, 67.4, 63.7, 55.0, 53.6, 24.2. LC-MS m/z calculated for C_17_H_21_NS_2_ [M+H]^+^ 302.10 found 302.14.

#### 3.1.5. Synthesis of 2-(dibenzylamino)-2-methylpropane-1,3-diol (**3b**)

Anhydrous K_2_CO_3_ (4 g, 29.48 mmol) was added to a stirring solution of **2b** (1 g, 9.51 mmol), in EtOH (50 mL). Benzyl bromide (3.50 mL, 29.48 mmol) was then added dropwise to the solution with stirring. The resultant mixture was refluxed for 16 h. After completion of the reaction, the mixture was cooled to room temperature, and volatiles were removed under reduced pressure. The residue was quenched with saturated NaHCO_3_ and extracted with EtOAc (3 × 20 mL). The organic layer was dried over anhydrous MgSO_4_ and filtered. The solvent was then removed under reduced pressure to afford a solid crude product, which was triturated with *n-*hexane to result in the title compound as a white solid (2.5 g, 92%). HPLC *t*_R_ = 6.07 min; ^1^H NMR (400 MHz, DMSO-*d_6_*, 25 °C) *δ* 7.24 (d, *J* = 7.90 Hz, 4H), 7.15 (t, *J* = 7.15 Hz, 4H), 7.06 (t, *J* = 7.15 Hz, 2H), 4.34 (t, *J* = 5.05 Hz, 2H), 3.84 (s, 4H), 3.48–3.40 (m, 4H), 0.98 (s, 3H). ^13^C NMR (100 MHz, DMSO-*d_6_*, 25 °C) *δ* 142.5, 127.9, 127.6, 125.9, 64.2, 62.4, 53.5, 17.4. LC-MS m/z calculated for C_18_H_23_NO_2_ [M+H]^+^ 286.17 found 286.22.

#### 3.1.6. Synthesis of S,S’-(2-(dibenzylamino)-2-methylpropane-1,3-diyl) diethanethioate (**4b**)

Excess SOCl_2_ was added to a stirring solution of **3b** (2 g, 7.0 mmol) in DCM (20 mL) under nitrogen. The resultant mixture was stirred at 60 °C and monitored by TLC until no starting material was observed. After completion of the reaction (8 h), the solvent was removed to result in a white semi-solid residue. This residue was dissolved in ACN and a solution of potassium thioacetate (4 g, 35.03 mmol, 5 eq.) in ACN with a few drops of DMF (for solubility) added, followed by TEA (0.09 mL, 0.7 mmol). The reaction was heated at 90 °C and monitored by TLC. After 20 h, when no starting material was observed, the solvent was removed under reduced pressure, diluted with H_2_O, and extracted with EtOAc (3 × 30 mL). The organic layer was dried over anhydrous MgSO_4_ and filtered. The solvent was then removed under reduced pressure to obtain a crude product, which was purified by column chromatography using 100–200 mesh silica gel. The desired product was eluted with 6% *v*/*v* ethyl acetate/hexane to present a red semi-solid title compound (1.25 g, 44%). HPLC *t*_R_ = 12.63 min; ^1^H NMR (400 MHz, DMSO-*d_6_*, 25 °C) *δ* 7.30 (d, *J* = 7.23 Hz, 4H), 7.19 (t, *J* = 7.66 Hz, 4H), 7.09 (t, *J* = 7.28 Hz, 2H), 3.78 (s, 4H), 3.35 (s, 1H), 3.32 (brs, 1H), 3.19 (s, 1H), 3.16 (s, 1H), 2.36 (s, 6H), 0.98 (s, 3H). ^13^C NMR (100 MHz, DMSO-*d_6_*, 25 °C) *δ* 195.1, 141.2, 128.1, 127.9, 126.4, 61.4, 52.8, 35.5, 30.6, 20.9. LC-MS m/z calculated for C_22_H_27_NO_2_S_2_ [M+H]^+^ 402.15 found 402.20.

#### 3.1.7. Synthesis of 2-(dibenzylamino)-2-methylpropane-1,3-dithiol (**1b**)

Anhydrous HCl/MeOH (5 mL) was added to **4b** (0.15 g, 0.37 mmol) under nitrogen. The resultant mixture was refluxed for 2 h under nitrogen. After completion of the reaction, the mixture was cooled to room temperature and concentrated under reduced pressure, followed by azeotrope with toluene (3 × 15 mL) to afford a semi-solid residue, which was stored in vacuum for 4 h to dryness to obtain light yellow solid **1b** (DMPDT) (0.090 g, 95%). HPLC *t*_R_ = 8.61 min; ^1^H NMR (400 MHz, MeOD-*d_4_*, 25 °C) *δ* 7.40–7.37 (m, 4H), 7.36–7.32 (m, 2H), 7.26–7.23 (m, 4H), 4.87 (brs, 2H), 4.52 (brs, 2H), 3.43 (s, 1H), 3.39 (s, 1H), 3.22 (s, 1H), 3.18 (s, 1H), 1.69 (s, 3H). ^13^C NMR (100 MHz, MeOD-*d_4_*, 25 °C) *δ* 132.0, 131.0, 130.6, 130.3, 56.1, 29.8, 19.4. LC-MS m/z calculated for C_18_H_23_NS_2_ [M+H]^+^ 318.13 found 318.12.

#### 3.1.8. Synthesis of N,N-dibenzyl-4-methyl-1,2-dithiolan-4-amine (**1b**^OX^)

**1b** (0.015 g, 0.047 mmol) was diluted with DCM and washed with a 6 N NaOH solution. The organic layer was dried over anhydrous MgSO_4_ and filtered. The solvent was removed under reduced pressure to afford DMPDT^OX^ as a light yellow solid (0.013 g, 93%). HPLC *t*_R_ = 8.27 min; ^1^H NMR (400 MHz, CDCl_3_, 25 °C) *δ* 7.35–7.33 (m, 2H), 7.28–7.26 (m, 4H), 7.21–7.20 (m, 2H), 7.16–7.14 (m, 2H), 3.86 (s, 4H), 3.31 (s, 1H), 3.29 (s, 1H), 2.32 (s, 1H), 2.89 (s, 1H), 1.50 (s, 3H).^13^C NMR (100 MHz, CDCl_3_, 25 °C) *δ* 140.8, 128.3, 128.2, 126.9, 75.6, 55.9, 48.4, 23.6. LC-MS m/z calculated for C_18_H_21_NS_2_ [M+H]^+^ 316.11 found 316.16.

#### 3.1.9. General Procedure for the Study of DPDT as a Reducing Agent in Solution Phase for Fmoc-Cys(SDMP)-OH

The equimolar solution (10 mmol) of DPDT and Fmoc-Cys(SDMP)-OH was prepared separately in ACN. To study the reduction reaction, different ratios (1:1, 1:2, and 1:3) of the two solutions were prepared. For DIEA: H_2_O was added to this solution to obtain a final composition as the above solution/DIEA/H_2_O as 95:2.5:2.5. This was thoroughly mixed and 2 µL was immediately injected into HPLC to analyze the reducing strength.

### 3.2. General Procedure for the Synthesis of Peptide

Fmoc-rink amide AM resin (0.74 mmol/g, 1 eq.) was washed with DMF (3 × 1 min), DCM (3 × 1 min), and DMF (3 × 1 min). The Fmoc group was deprotected by treating the resin with 20% piperidine/DMF (1 × 1 min and 1 × 7 min) followed by washing with DMF, DCM, and DMF. The protected Fmoc-amino acids (3 eq.) were incorporated using DIC (3 eq.) and OxymaPure (3 eq.) in DMF for 30 min at rt. This process was repeated until the final peptide was obtained. DMF (3 × 1 min), DCM (3 × 1 min), and DMF (3 × 1 min) were used to wash between couplings and deprotections. Fmoc was removed from one peptide and protected with 3,3′-dithiodipropionic acid (DTDP; 3 eq.) using DIC (3 eq.) and OxymaPure (3 eq.) in DMF for 30 min. The resin was then washed as explained above and dried. Microcleavage was performed by treatment with TFA/TIS/H_2_O (95:2.5:2.5) for 1 h at room temperature. The cleavage mixture was evaporated with a stream of nitrogen, precipitated with Et_2_O, and centrifuged. The pellet was re-dissolved in H_2_O/ACN (1:1) for analysis by HPLC and LCMS.

### 3.3. General Procedure for the Study of DPDT as a Reducing Agent in Solid Phase

An amount of 5 mg of resin was washed in DMF. A solution of 15 eq. of DPDT in 600 µL of DMF was then prepared. DPDT/DIEA/H_2_O (95:2.5:2.5; 200 µL) was added to the washed resin. The supernatant was discarded after 10 min. The above reaction was repeated twice (10 min each). The resin was then washed with DMF and DCM and then dried under vacuum. Mini cleavage was performed for 1 h at rt using TFA/TIS/H_2_O (95:2.5:2.5). TFA vaporized with a stream of nitrogen, precipitated with Et_2_O, and centrifuged. The pellet was then re-dissolved in H_2_O/ACN (1:1) for HPLC and LCMS analysis.

## 4. Conclusions

In conclusion, here, we synthesized two novel dithiol-reducing agents, namely 2-(dibenzylamino) propane-1,3-dithiol (DPDT) and 2-(dibenzylamino)-2-methylpropane-1,3-dithiol (DMPDT), from inexpensive serinol and methyl serinol, respectively. We developed a highly convenient synthetic pathway with high-yielding steps that do not require time-consuming column chromatography and that are suitable for large-scale production (cost effective). DPDT was soluble in a variety of organic solvents, including DMSO, DMF, EtOH, MeOH, CHCl_3_, DCM, and ACN. Furthermore, we demonstrated the effectiveness of non-malodorous DPDT as a reducing agent in both liquid and solid phases. DPDT was observed to fully remove the SDMP- and SIT-protecting group from Cys thiol in solution and solid phase, respectively. A comparative study using DTT as a reducing agent in these two phases revealed that the reducing capacity of DPDT was comparable to that of DTT. Given these observations, DPDT emerges as an inexpensive alternative disulfide-reducing agent to DTT in peptide chemistry.

## Data Availability

The data presented in this study are available in this article or the Supplementary Materials.

## Supplementary Materials

The following supporting information can be downloaded at: https://www.mdpi.com/article/10.3390/molecules28145489/s1.

## Sample Availability

The data presented in this study are available in this article or Supplementary Material here.
