# Supplementary material for: Serinol-Based Versatile Disulfide-Reducing Reagent
Source: Molecules. 2023 Jul 18;28(14):5489. doi: 10.3390/molecules28145489 (PMC10384529; doi:10.3390/molecules28145489)
Supplement: Supplementary file 1 [file molecules-28-05489-s001.zip › molecules-2487153-supplementary.pdf]

# Serinol-Based Versatile Disulfide-Reducing Reagent †

**Babita Kushwaha [1], Sinenhlanhla N. Mthembu [1,2], Anamika Sharma [1,2,3], Fernando Albericio [2,4,*] and Beatriz G. de la Torre [1,*]**

[1] KwaZulu-Natal Research Innovation and Sequencing Platform (KRISP), School of Laboratory Medicine and Medical Sciences, College of Health Sciences, University of KwaZulu-Natal, Durban 4041, South Africa
[2] Peptide Science Laboratory, School of Chemistry and Physics, University of KwaZulu-Natal, Durban 4000, South Africa
[3] Department of Natural Products and Medicinal Chemistry, CSIR-Indian Institute of Chemical Technology, Hyderabad 500007, India
[4] CIBER-BBN, Networking Centre on Bioengineering, Biomaterials and Nanomedicine, Department of Organic Chemistry, University of Barcelona, 08028 Barcelona, Spain
* Correspondence: albericio@ukzn.ac.za (F.A.); garciadelatorreb@ukzn.ac.za (B.G.d.l.T.)
† Honoring the Nobel Laurate in Chemistry Professor Morten Meldal, who is a continuous inspiration for all of us.

## Supporting information

## Table of contents


| SI. No. | Contents | Page No. |
|---|---|---|
| **1.** | Copies of $^1$H, $^{13}$C, HPLC and Mass spectra of synthesized compounds…….. | **S2** |
| **2.** | Stability test of DPDT in solid form. (**A**) freshly prepared; **(B)** after 60 days.. | **S18** |
| **3.** | Stability test of DMPDT in solid form. (**A**) freshly prepared; **(B)** after 60 days. | **S19** |
| **4.** | HPLC chromatogram of Fmoc-Cys(SDMP)-OH with DTT (1:1 and 1:2 ratio) with 2.5% DIEA and 2.5% water at 0 min…………………………………….. | **S20** |
| **5.** | Effect of different bases in reduction of Fmoc-Cys(S-DMP)-OH using DPDT in ACN/base/$H_2O$ (95:2.5:2.5)……………………………………………… | **S21** |
| **6.** | Fmoc-Ala-Cys(SIT)-Leu-NH-resin after treatment with DTT with 2.5% DIEA and 2.5% water in DMF…………………………………………………….. | **S22** |

*1. Copies of $^{1}H$, $^{13}C$, HPLC and Mass spectra of synthesized compounds*

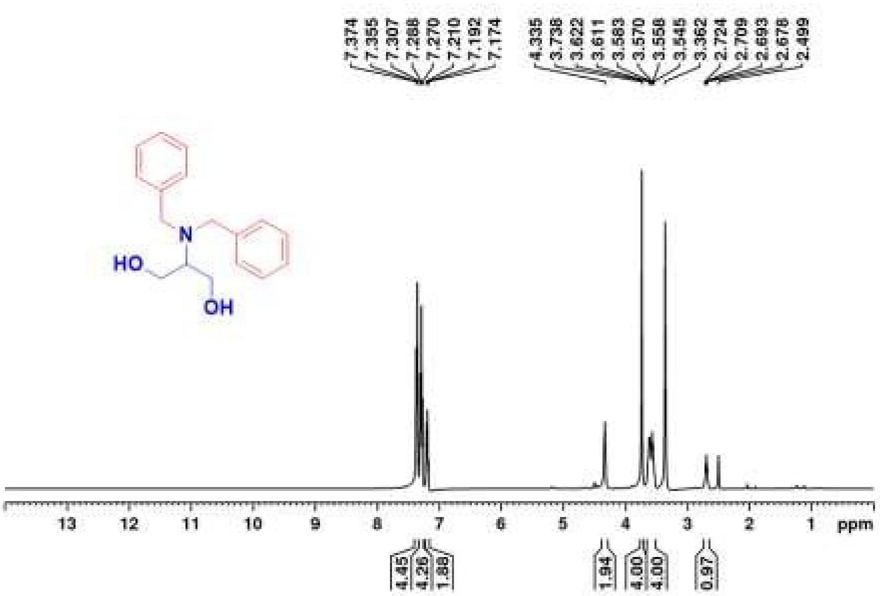


**Figure S1: $^{1}$H NMR of Compound 3a (400 MHz, DMSO-$d_6$)**

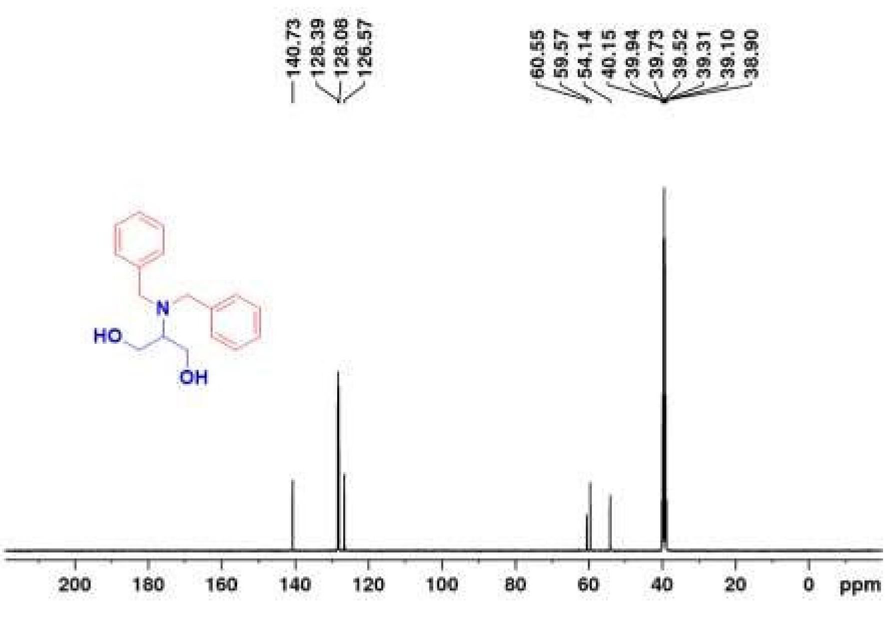


**Figure S2: $^{13}$C NMR of Compound 3a (100 MHz, DMSO-$d_6$)**

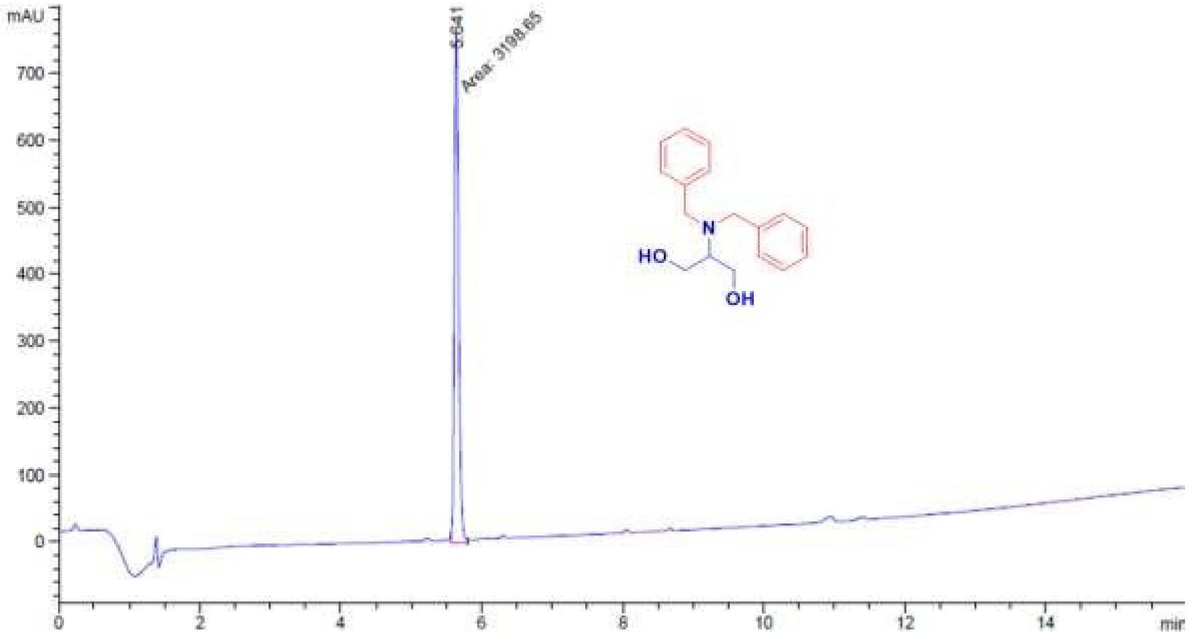


**Figure S3: HPLC of Compound 3a**

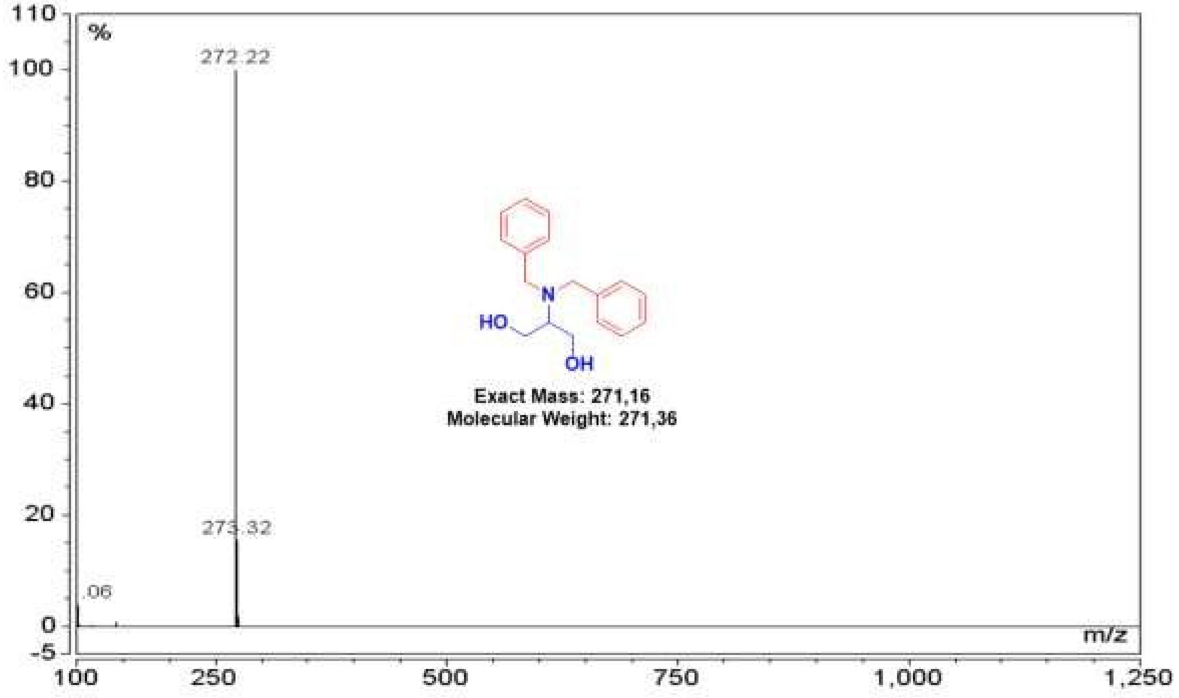


**Figure S4: Mass of Compound 3a**

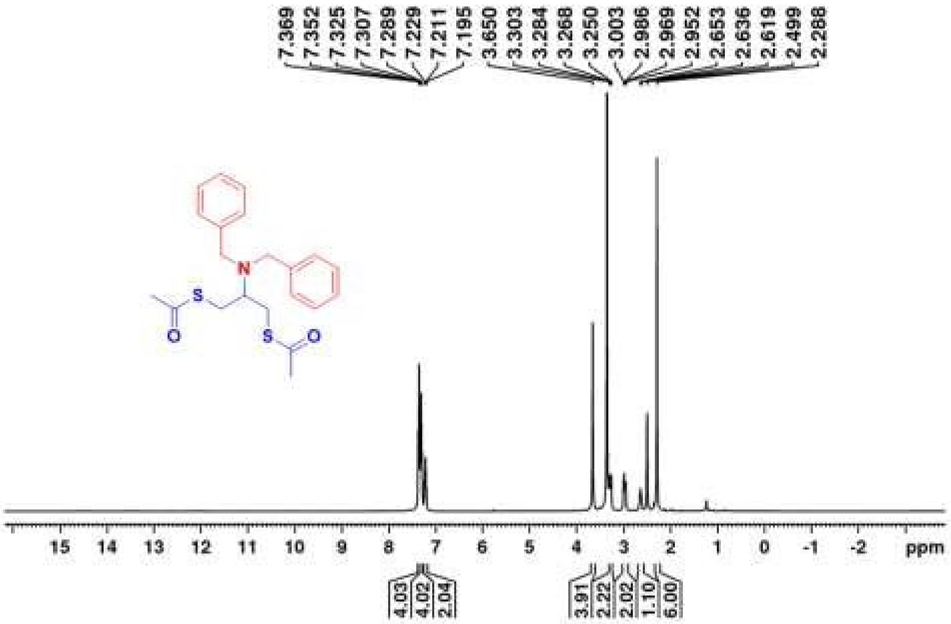


Figure S5: $^{1}$H NMR of Compound 4a (400 MHz, DMSO-$d_6$)

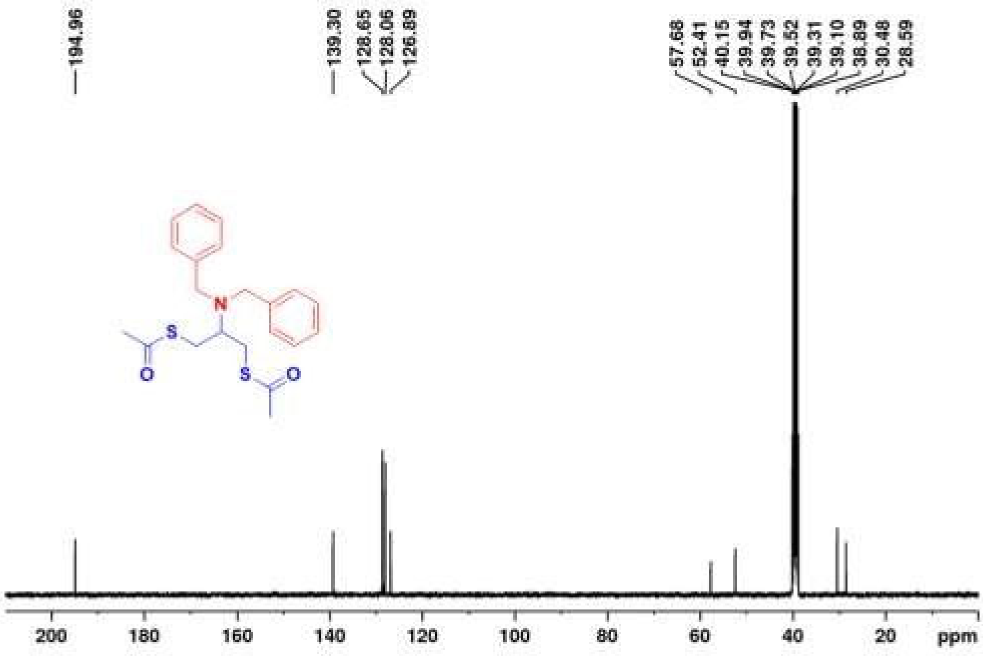


Figure S6: $^{13}$C NMR of Compound 4a (100 MHz, DMSO-$d_6$)

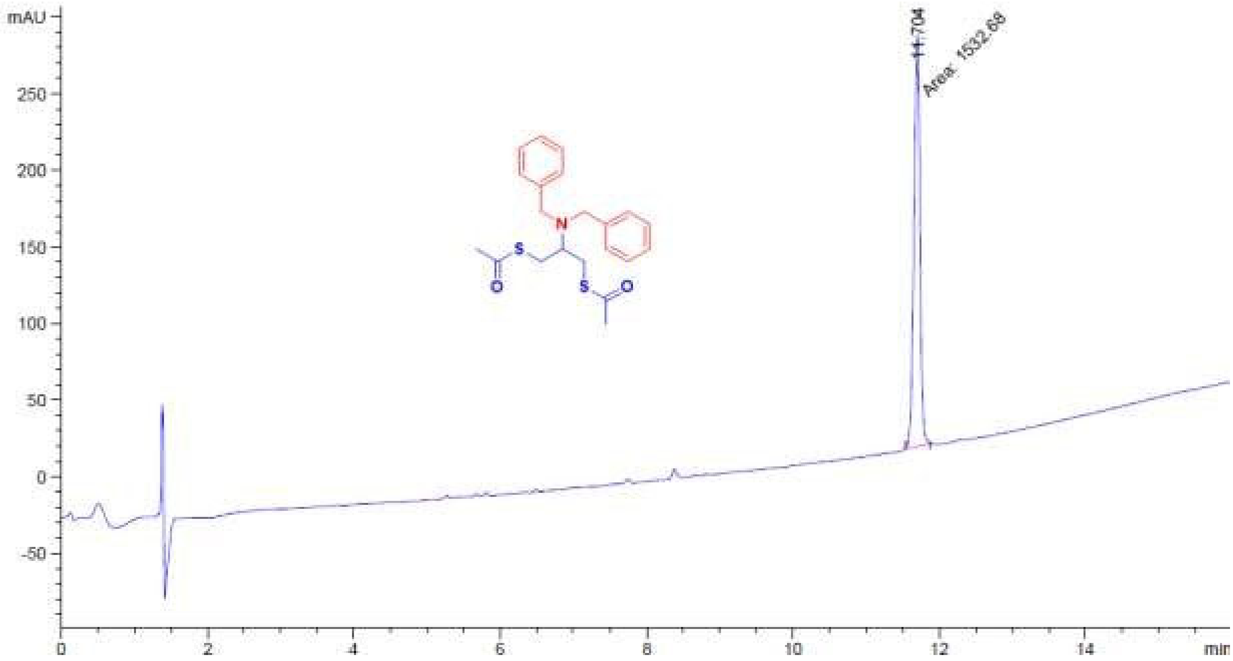


Figure S7: HPLC of Compound 4a

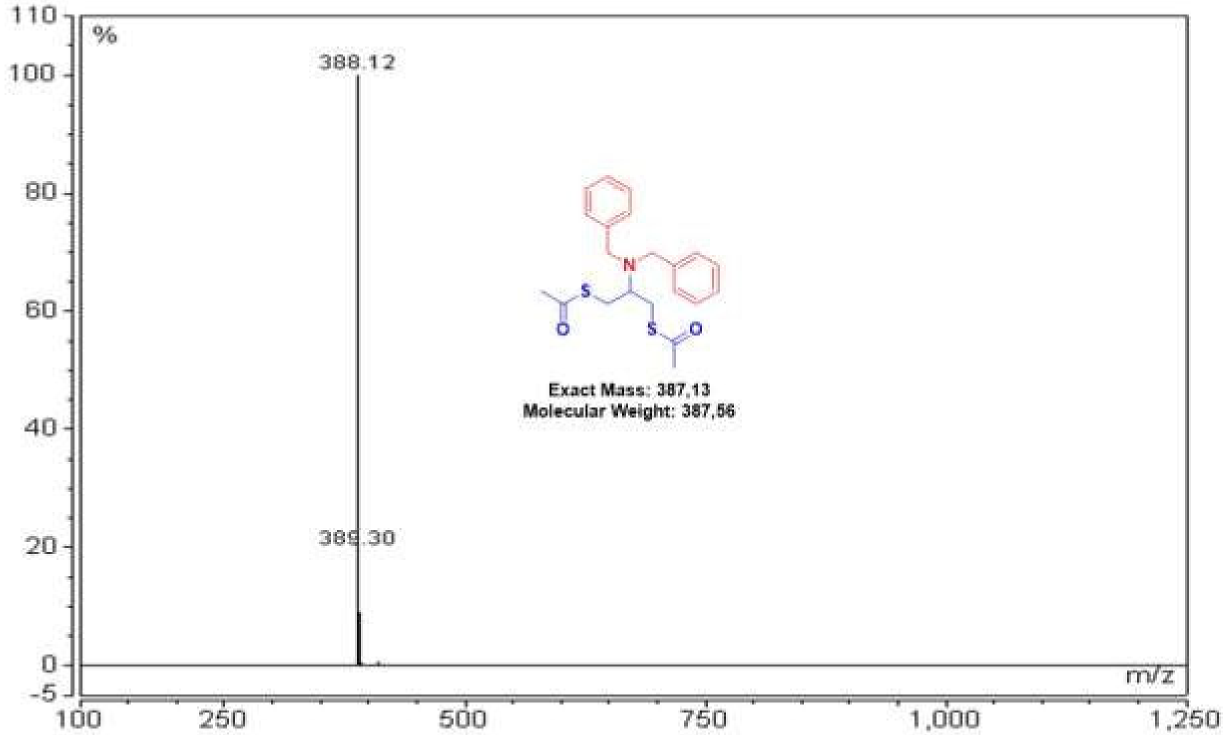


Figure S8: Mass of Compound 4a

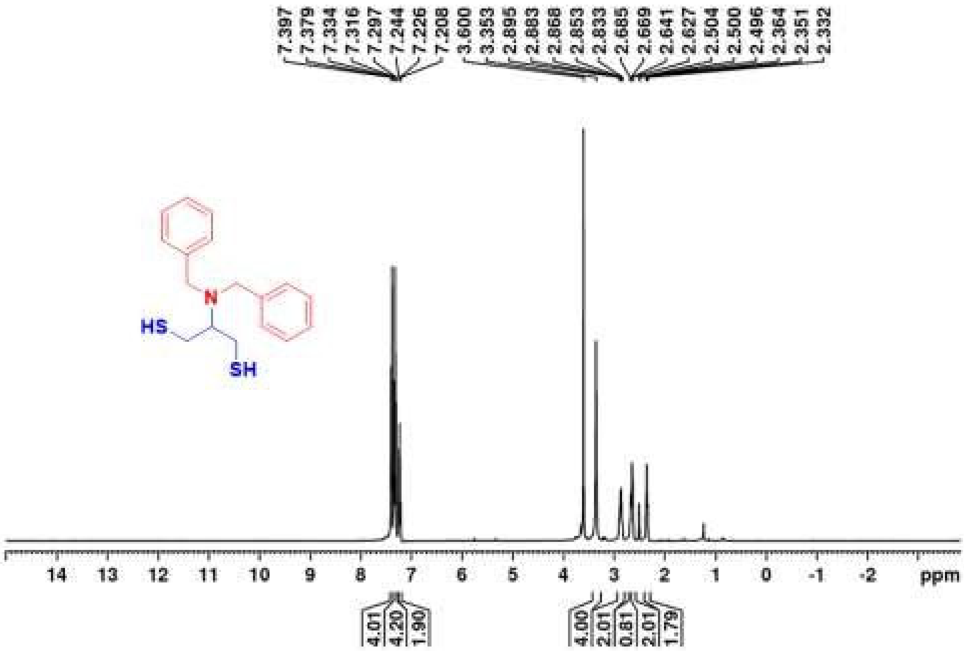


**Figure S9: $^{1}$H NMR of Compound 1a (DPDT) (400 MHz, DMSO-$d_6$)**

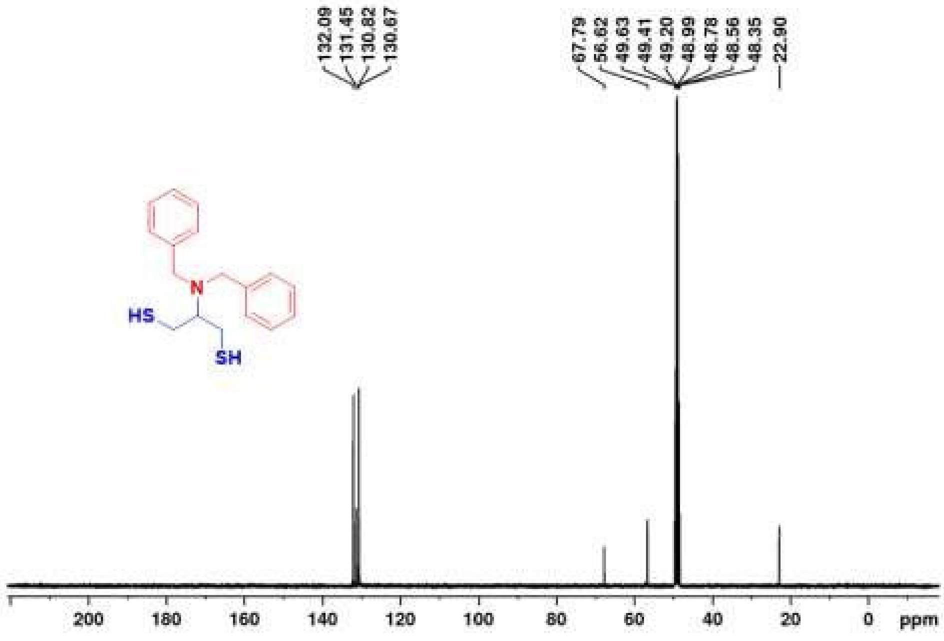


**Figure S10: $^{13}$C NMR of Compound 1a (DPDT) (100 MHz, MeOD-$d_4$)**

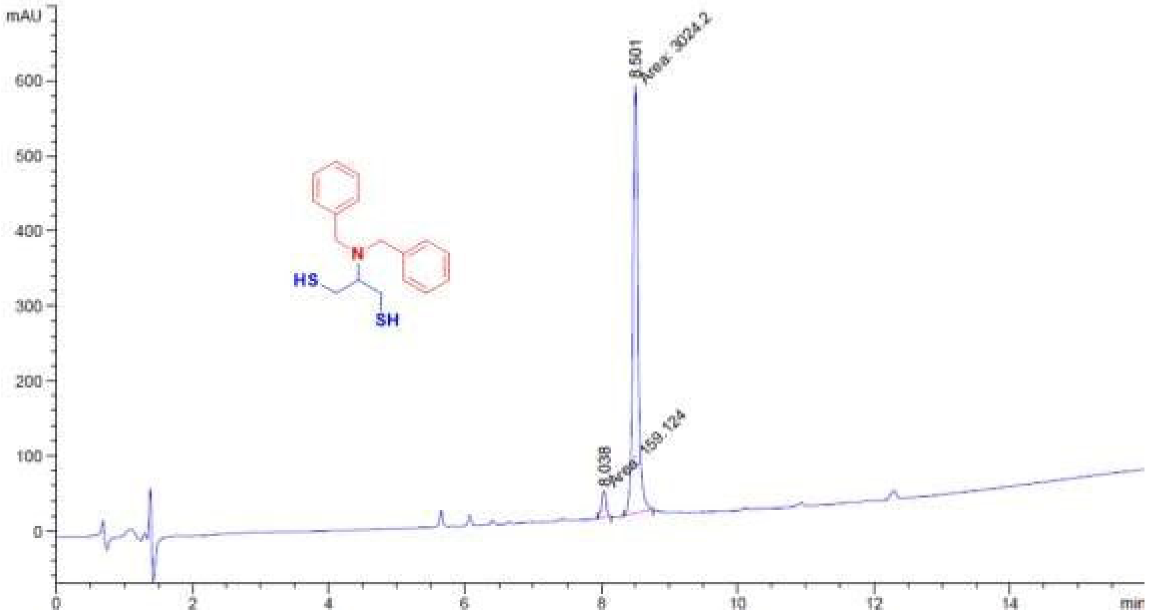


**Figure S11: HPLC of Compound 1a (DPDT)**

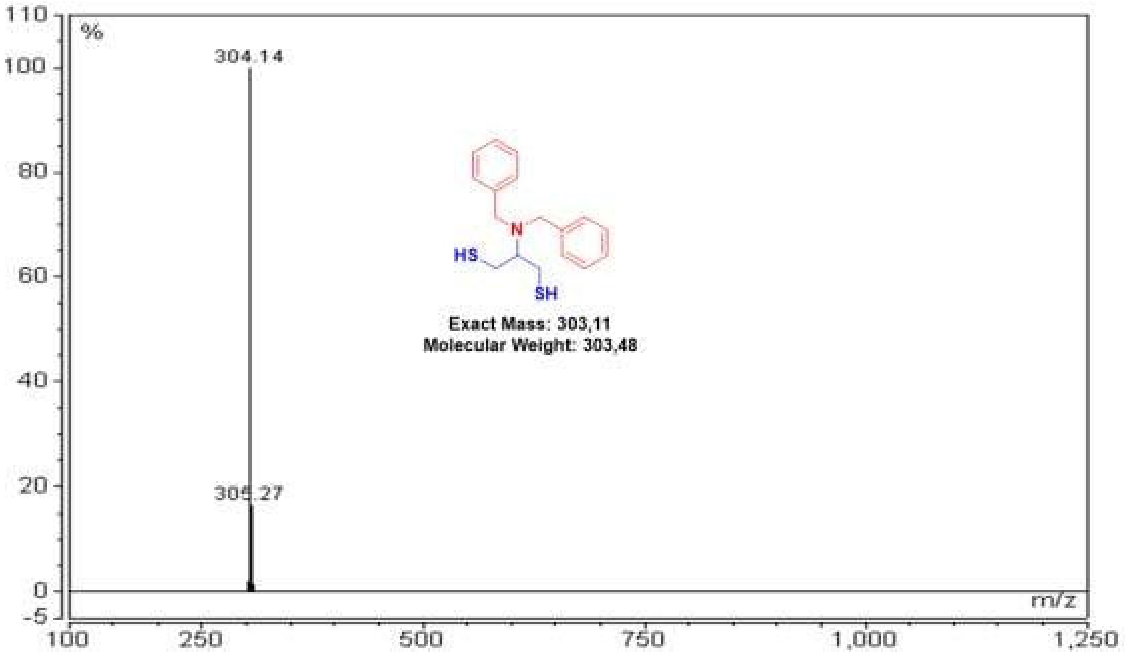


**Figure S12: Mass of Compound 1a (DPDT)**

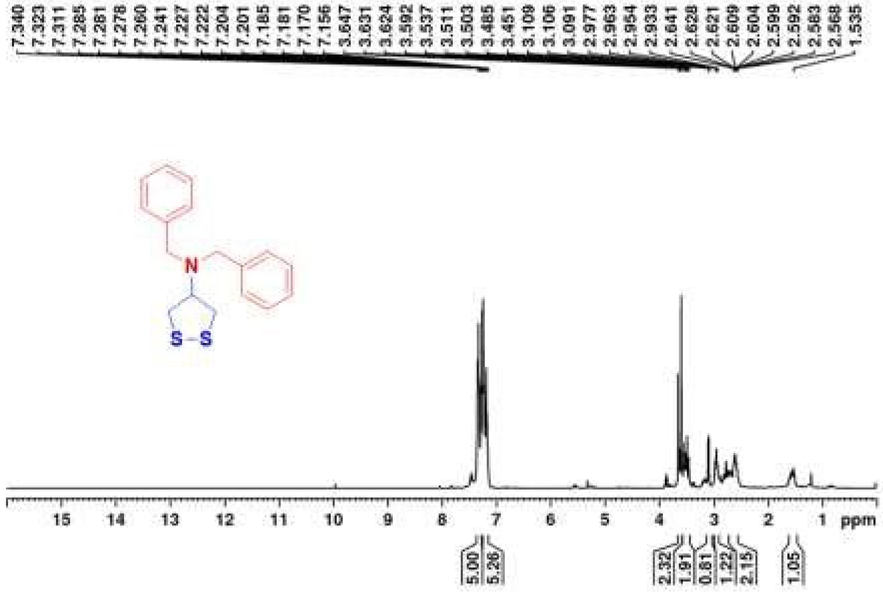


**Figure S13: $^{1}$H NMR of Compound 1a$^{OX}$ (DPDT$^{OX}$) (400 MHz, $CDCl_3$)**

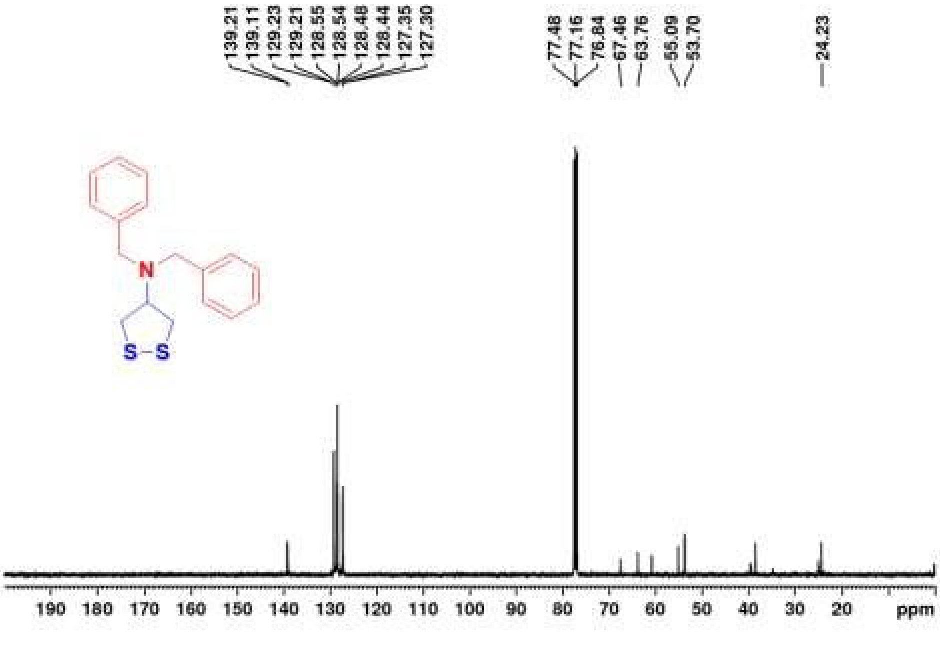


**Figure S14: $^{13}$C NMR of Compound 1a$^{OX}$ (DPDT$^{OX}$) (100 MHz, $CDCl_3$)**

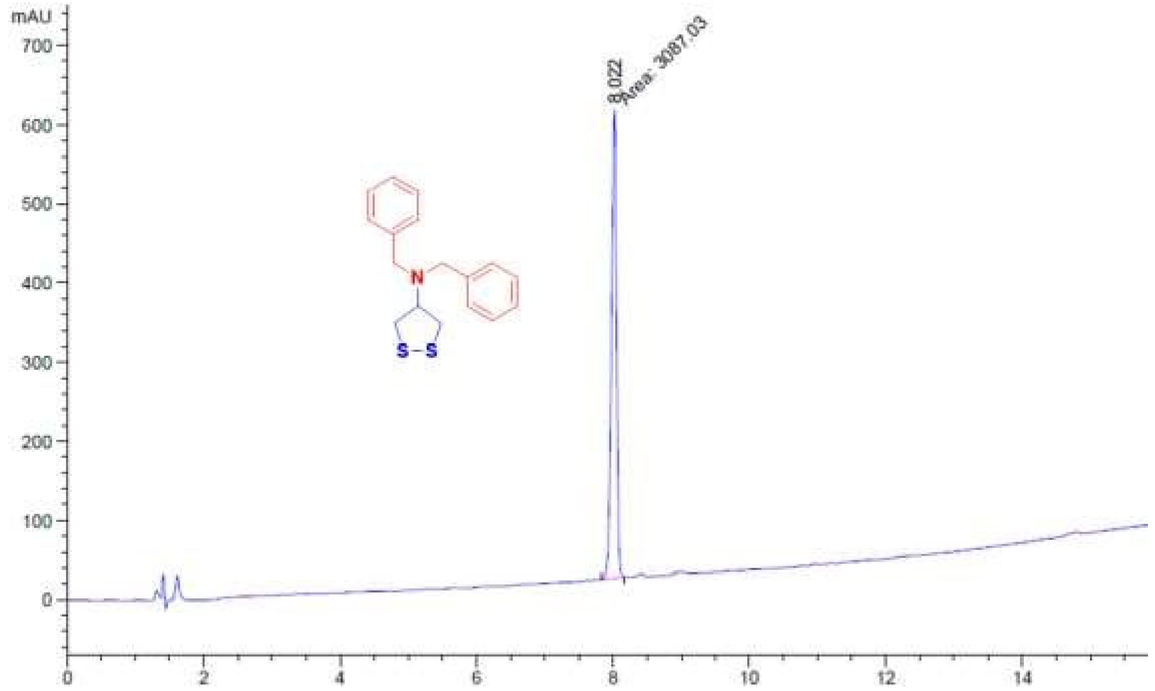


**Figure S15: HPLC of Compound 1a$^{OX}$ (DPDT$^{OX}$)**

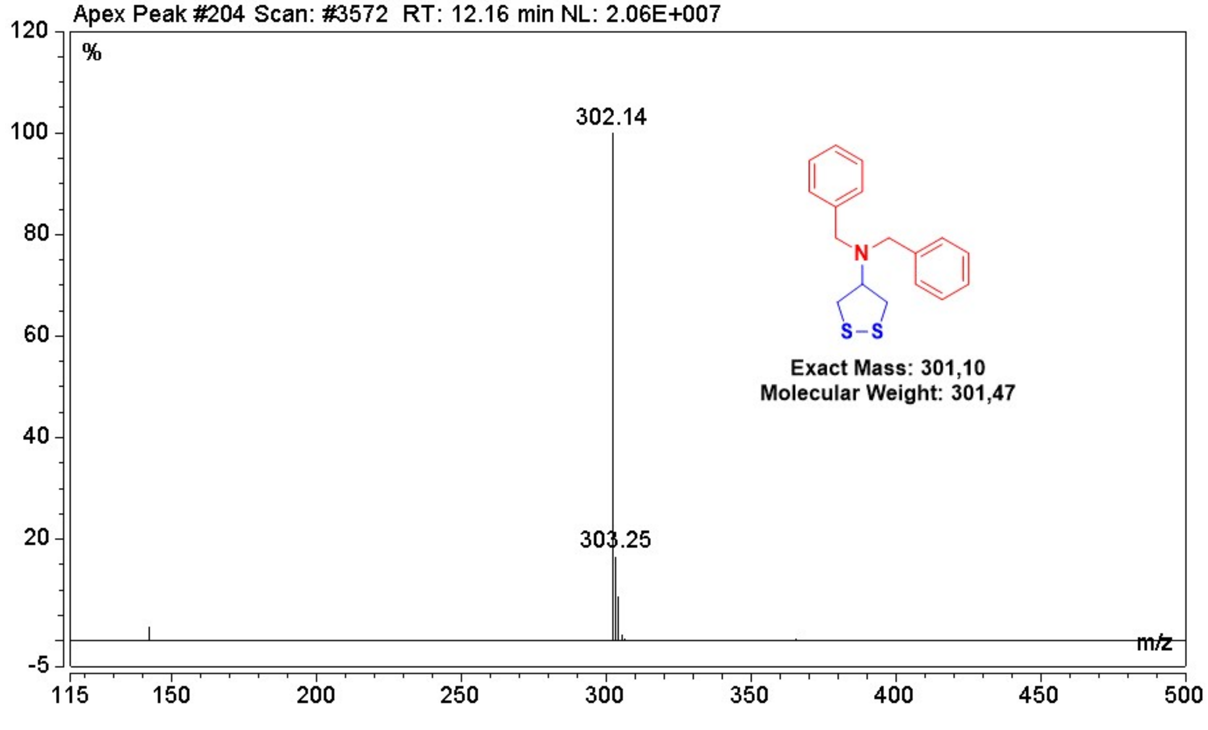


**Figure S16: Mass of Compound 1a$^{OX}$ (DPDT$^{OX}$)**

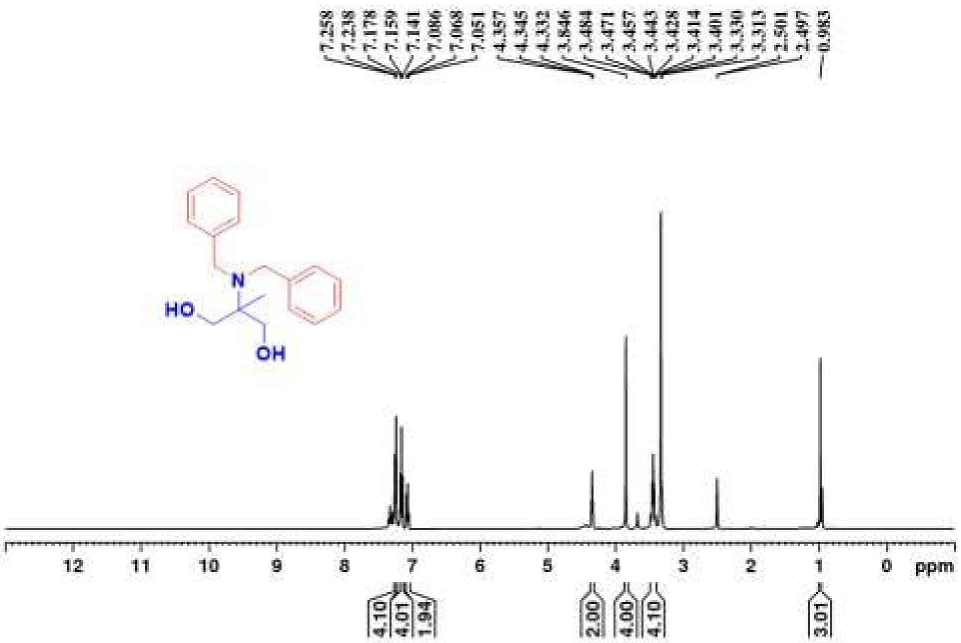


**Figure S17: $^{1}$H NMR of Compound 3b (400 MHz, DMSO-*d*$_6$)**

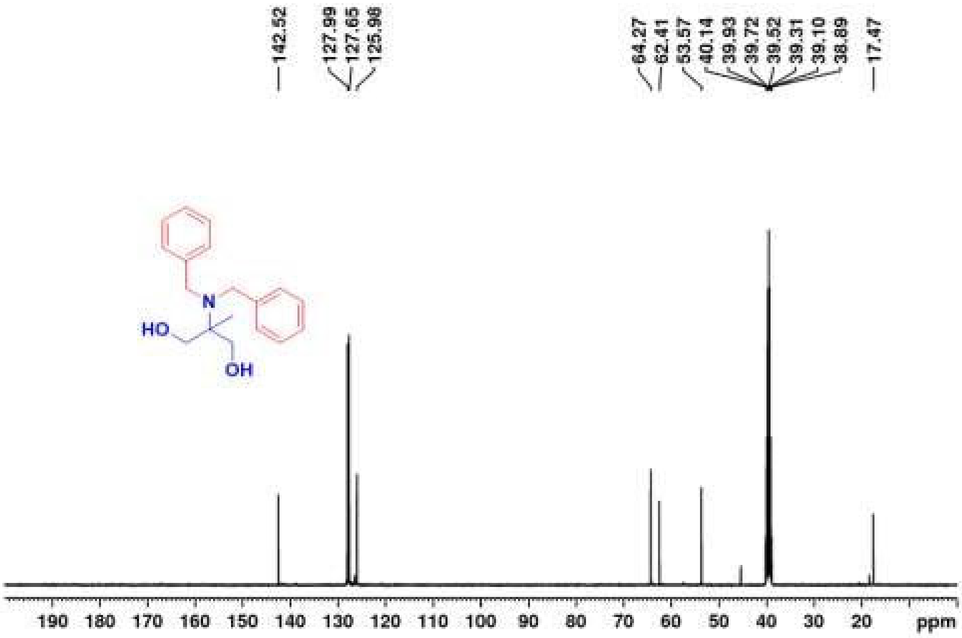


**Figure S18: $^{13}$C NMR of Compound 3b (100 MHz, DMSO-*d*$_6$)**

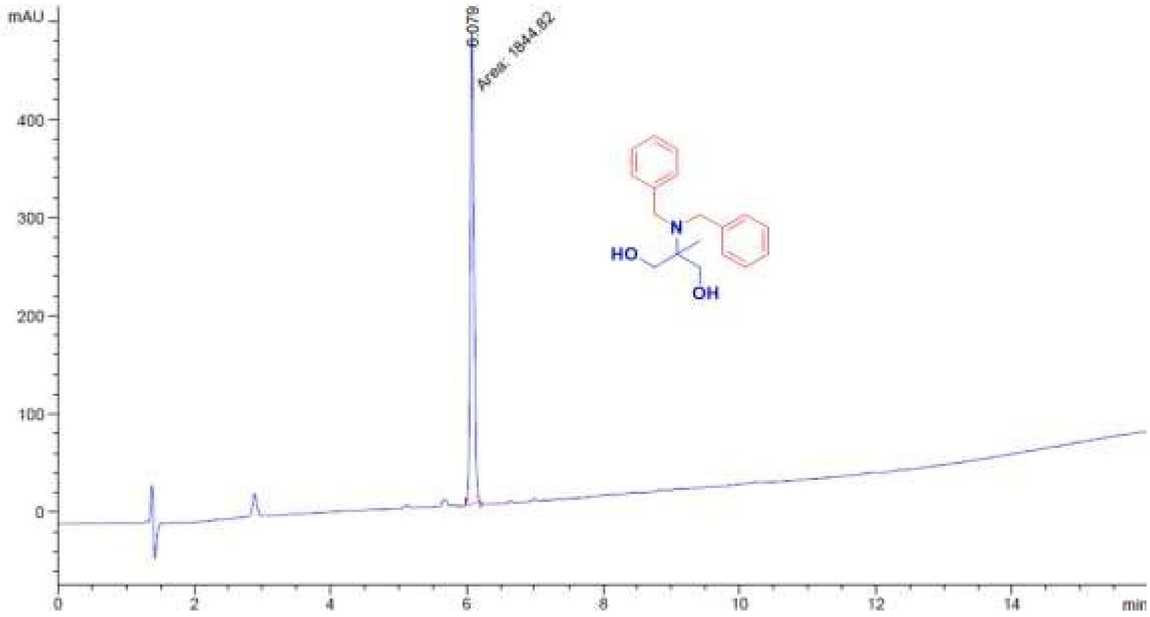


**Figure S19: HPLC of Compound 3b**

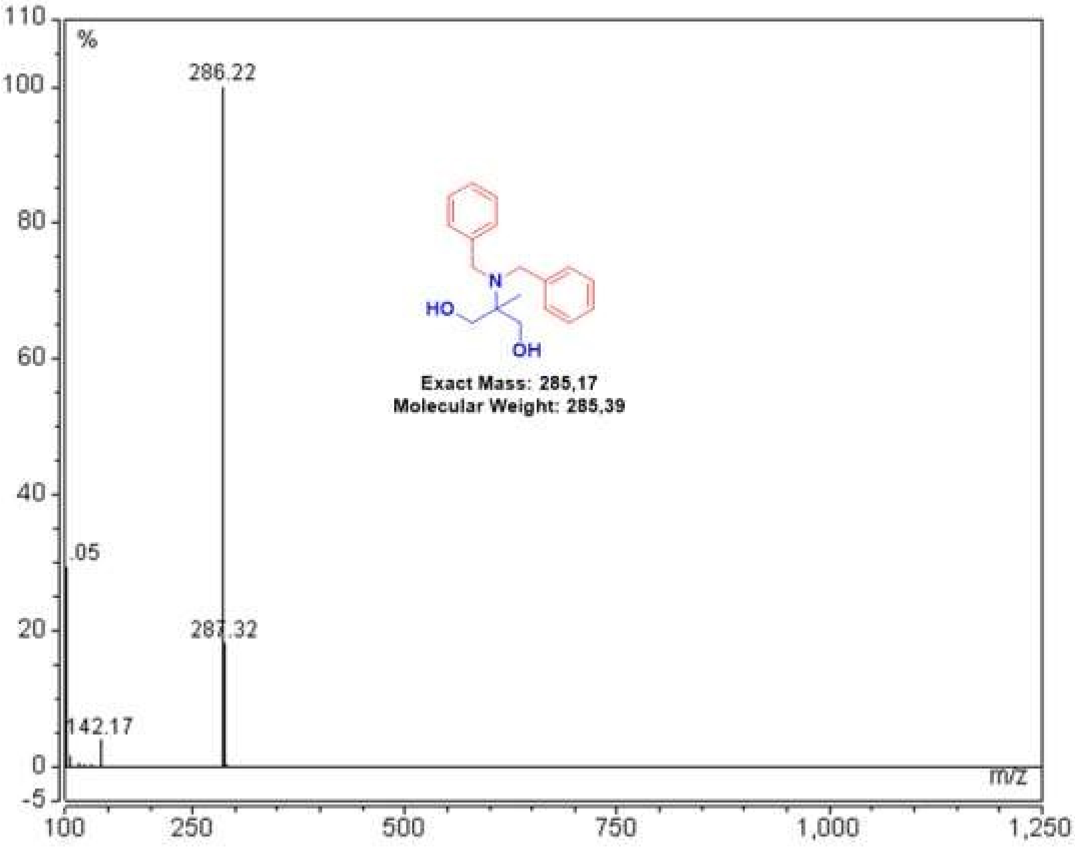


**Figure S20: Mass of Compound 3b**

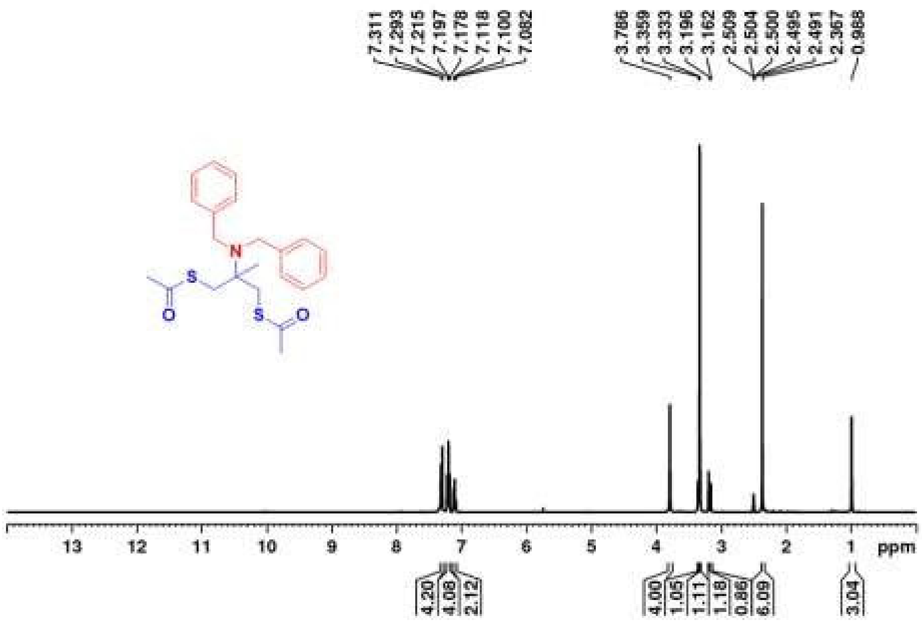


**Figure S21: $^{1}$H NMR of Compound 4b (400 MHz, DMSO-$d_6$)**

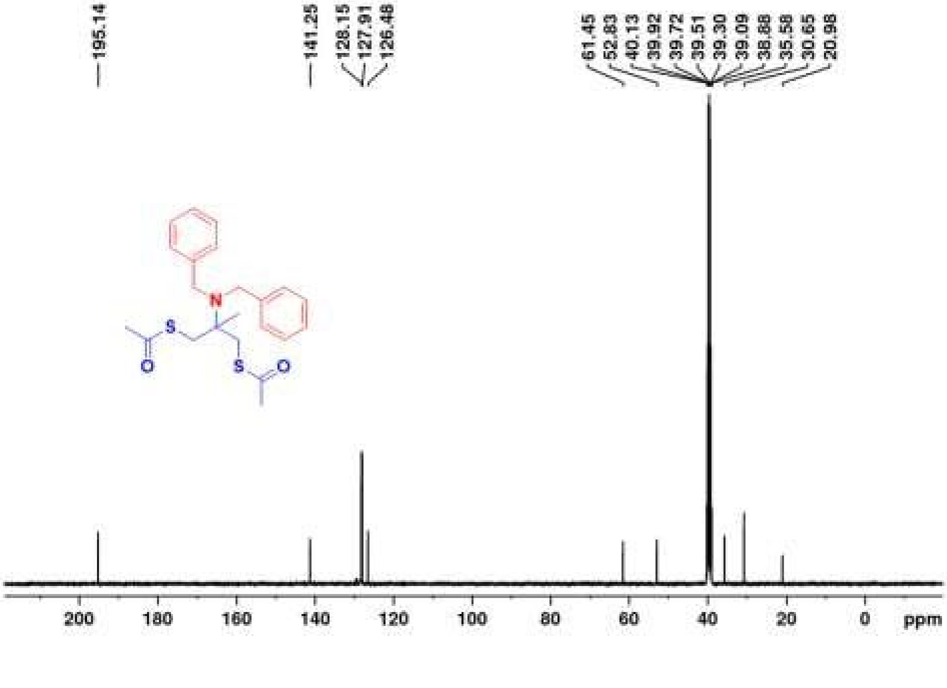


**Figure S22: $^{13}$C NMR of Compound 4b (100 MHz, DMSO-$d_6$)**

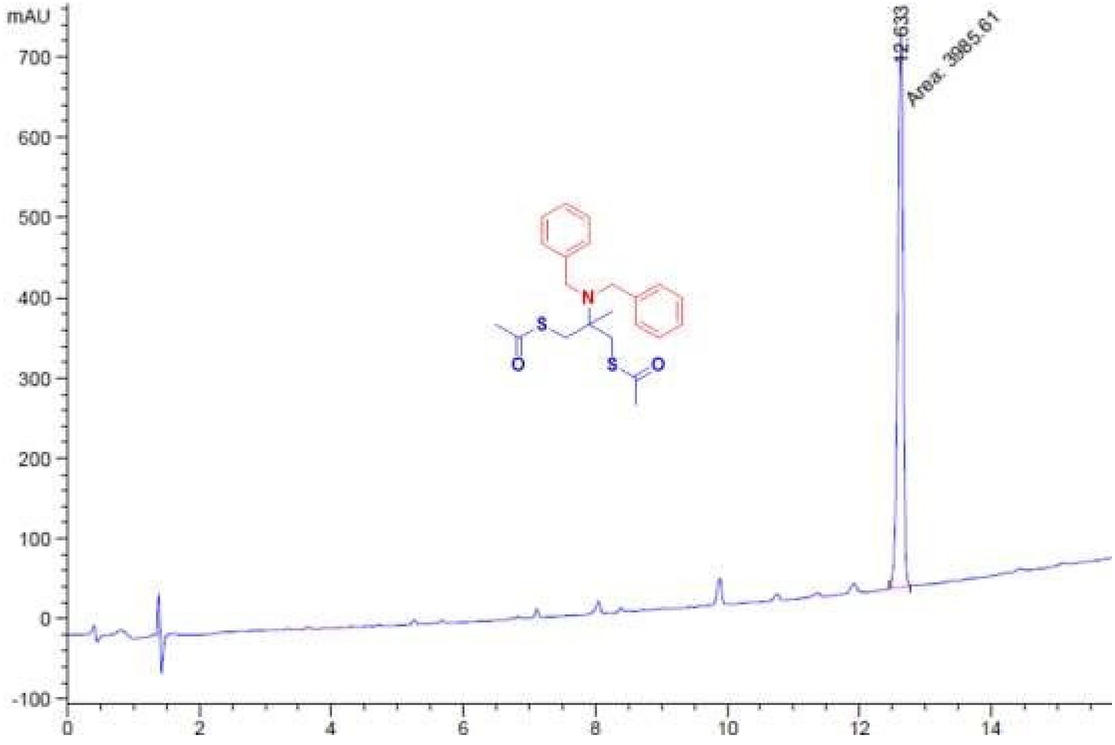


**Figure S23: HPLC of Compound 4b**

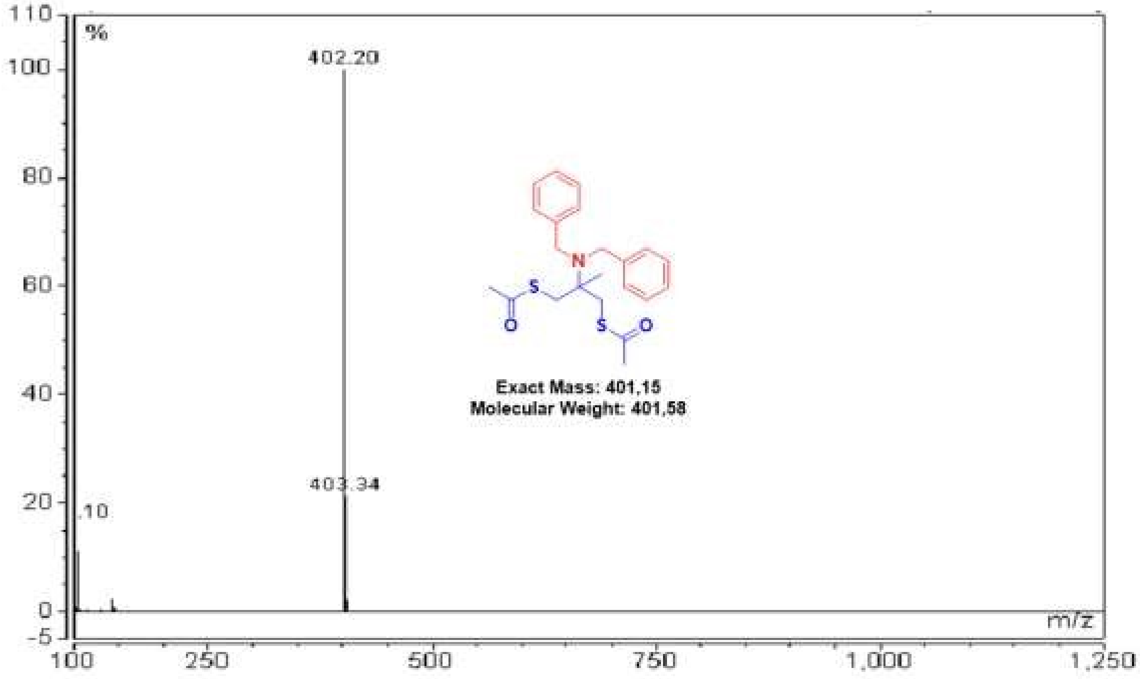


**Figure S24: Mass of Compound 4b**

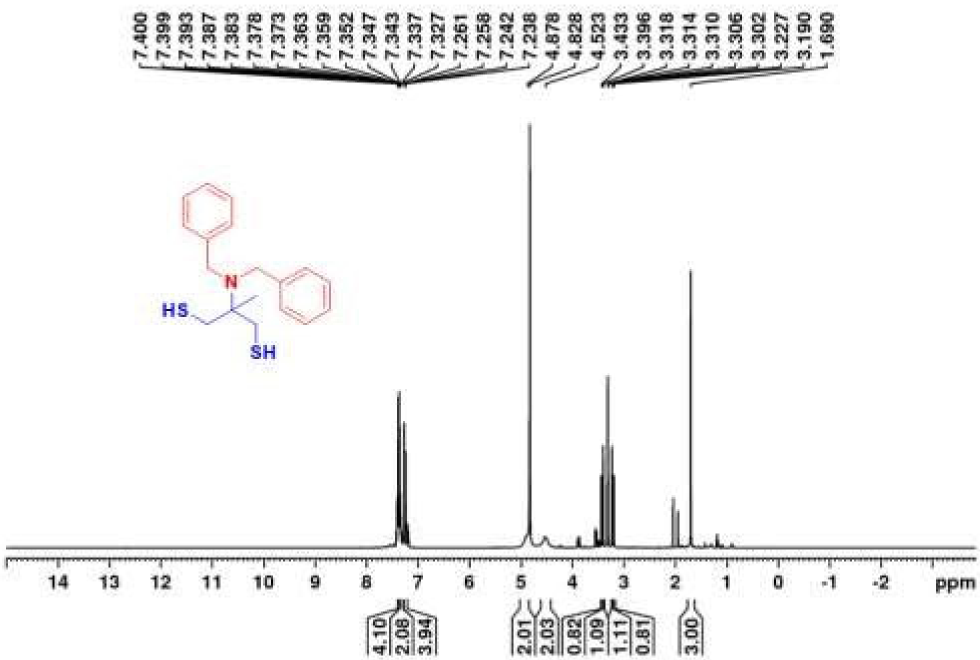


**Figure S25: $^{1}$H NMR of Compound 1b (DMPDT) (400 MHz, MeOD-*d₄*)**

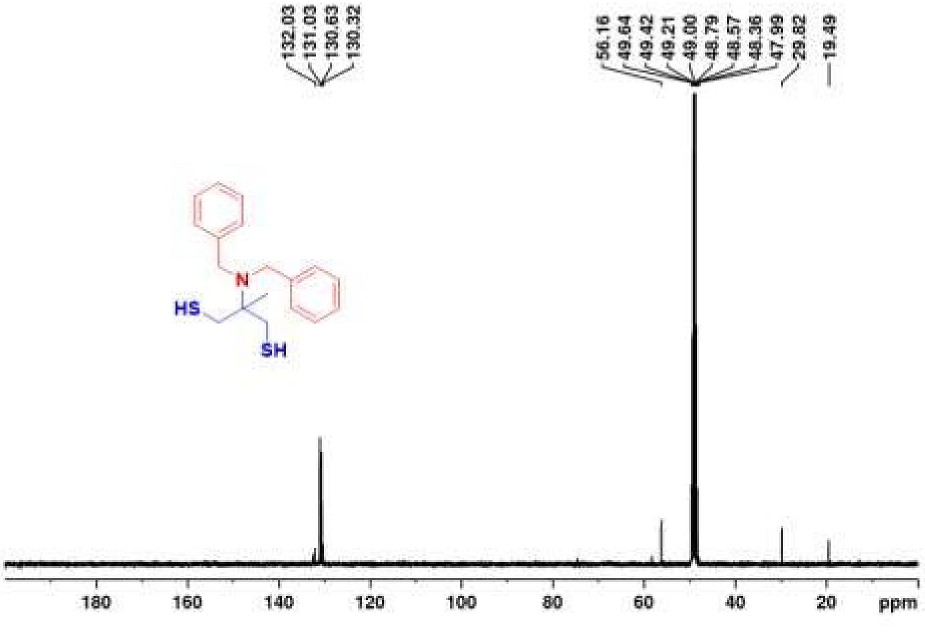


**Figure S26: $^{13}$C NMR of Compound 1b (DMPDT) (100 MHz, MeOD-*d₄*)**

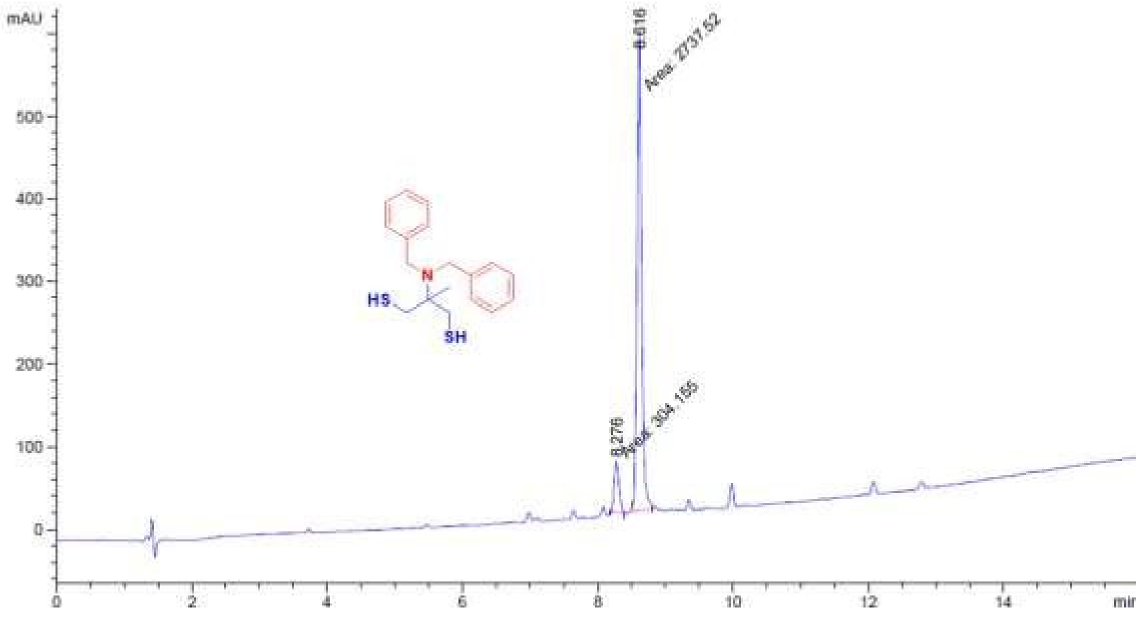


**Figure S27: HPLC of Compound 1b (DMPDT)**

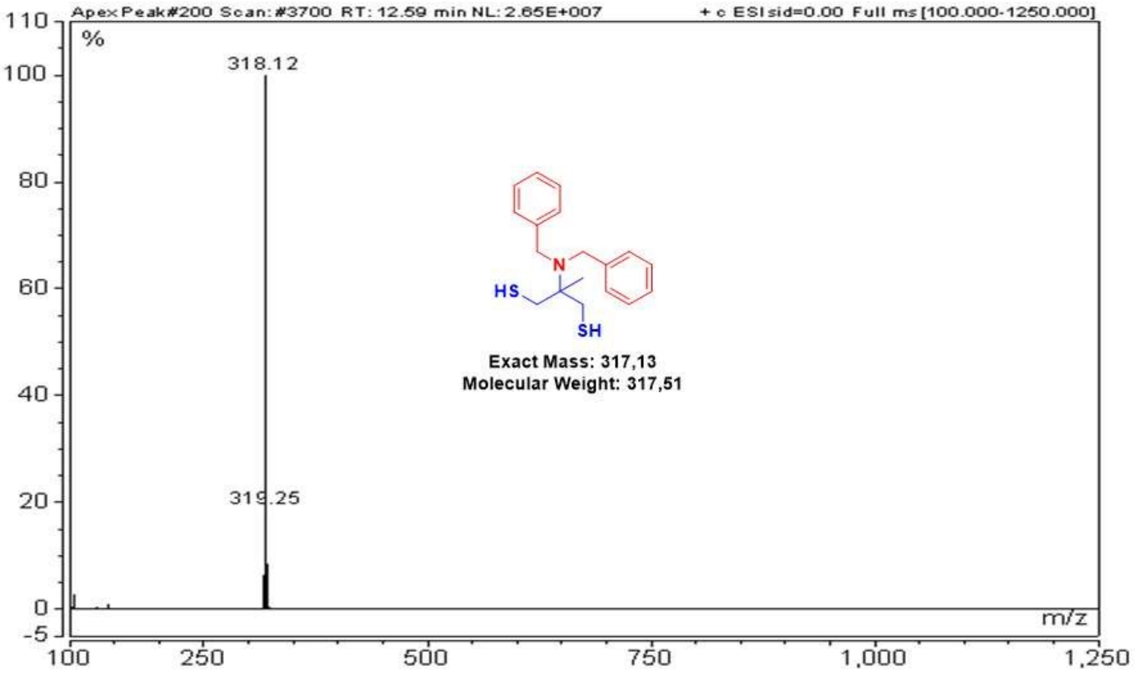


**Figure S28: Mass of Compound 1b (DMPDT)**

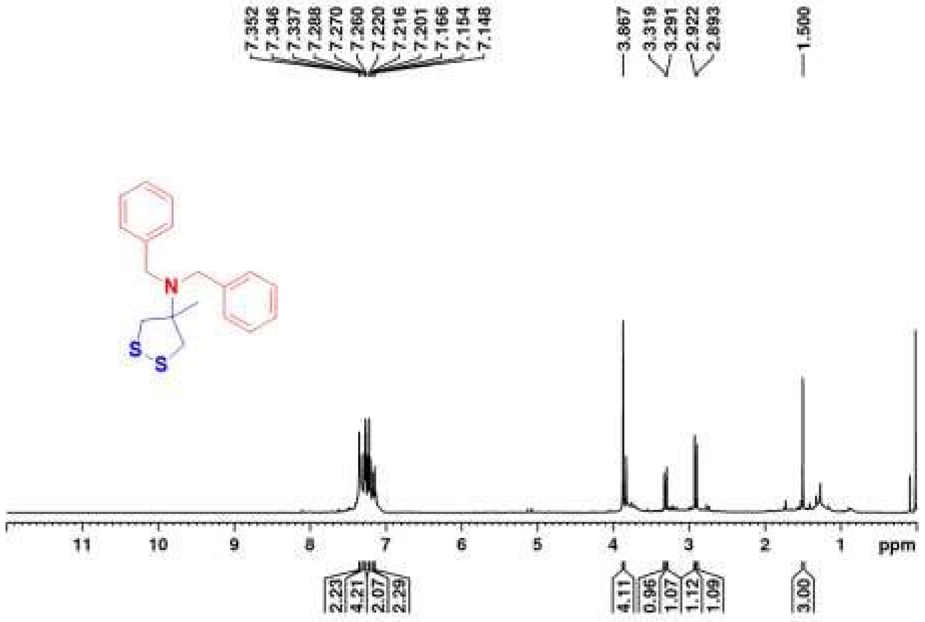


**Figure S29: $^{1}H$ NMR of Compound 1b$^{OX}$ (DMPDT$^{OX}$) (400 MHz, $CDCl_3$)**

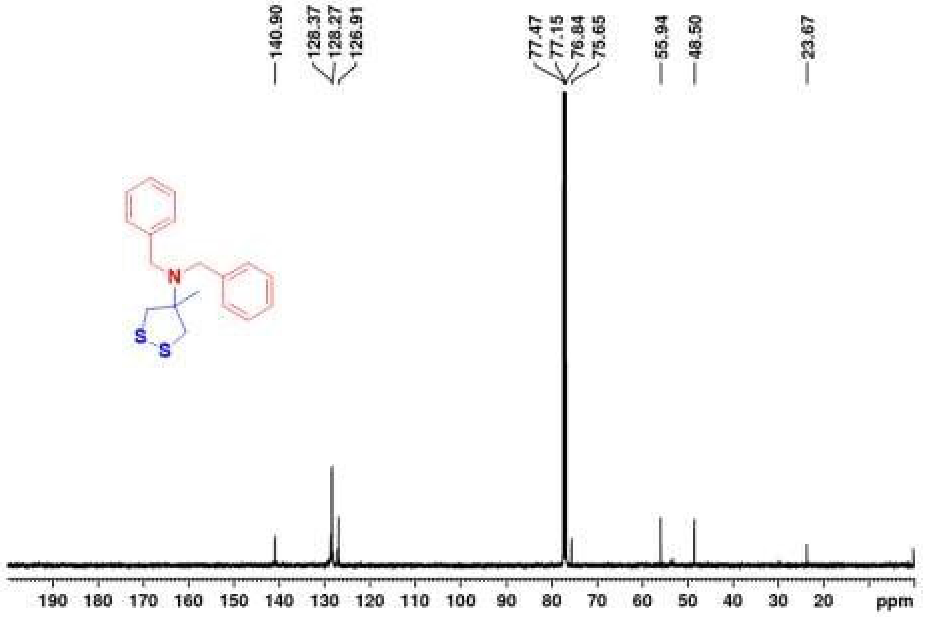


**Figure S30: $^{13}C$ NMR of Compound 1b$^{OX}$ (DMPDT$^{OX}$) (100 MHz, $CDCl_3$)**

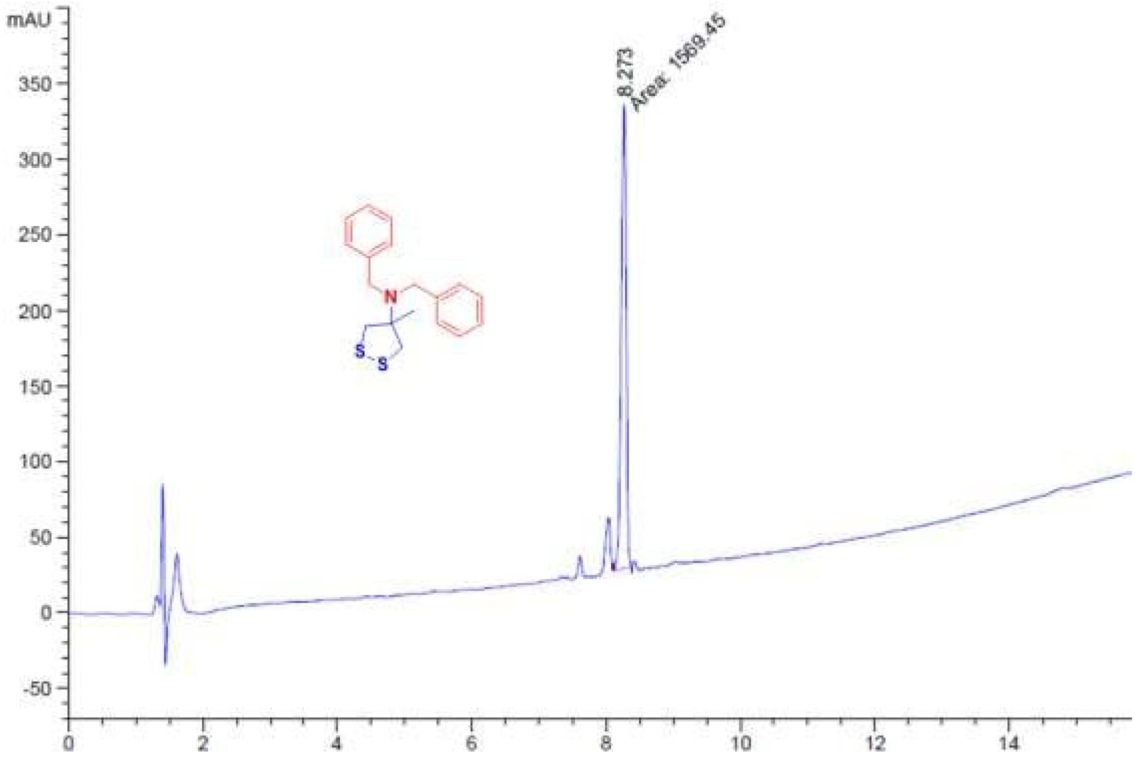


**Figure S31: HPLC of Compound 1b$^{OX}$ (DMPDT$^{OX}$)**

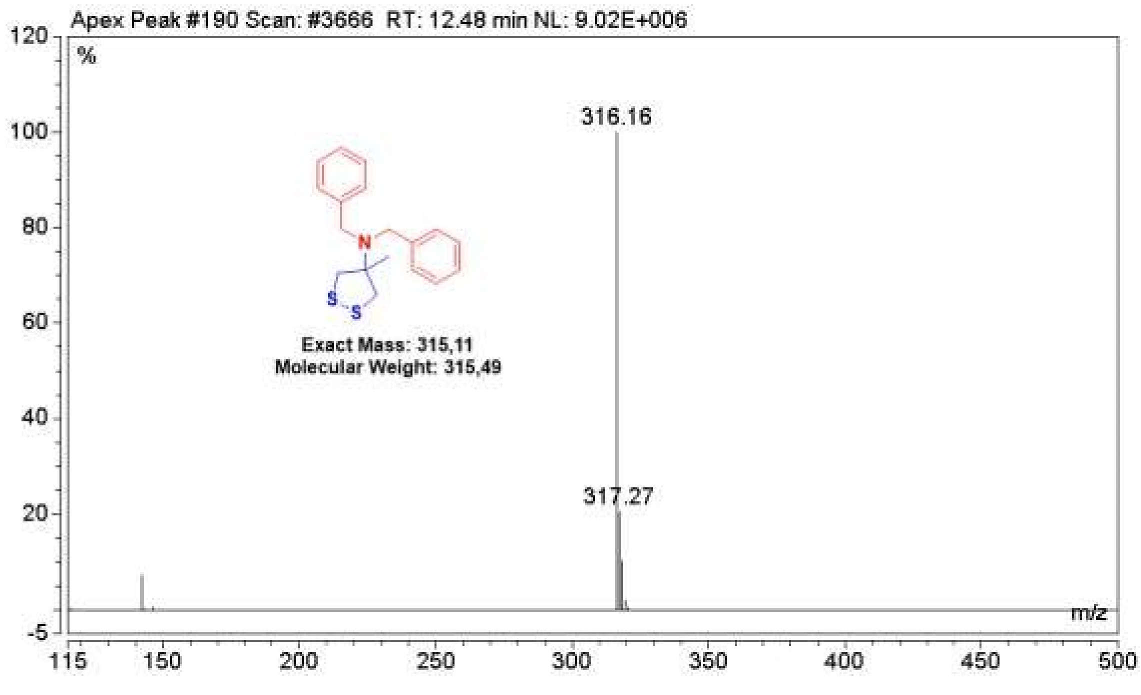


**Figure S32: Mass of Compound 1b$^{OX}$ (DMPDT$^{OX}$)**

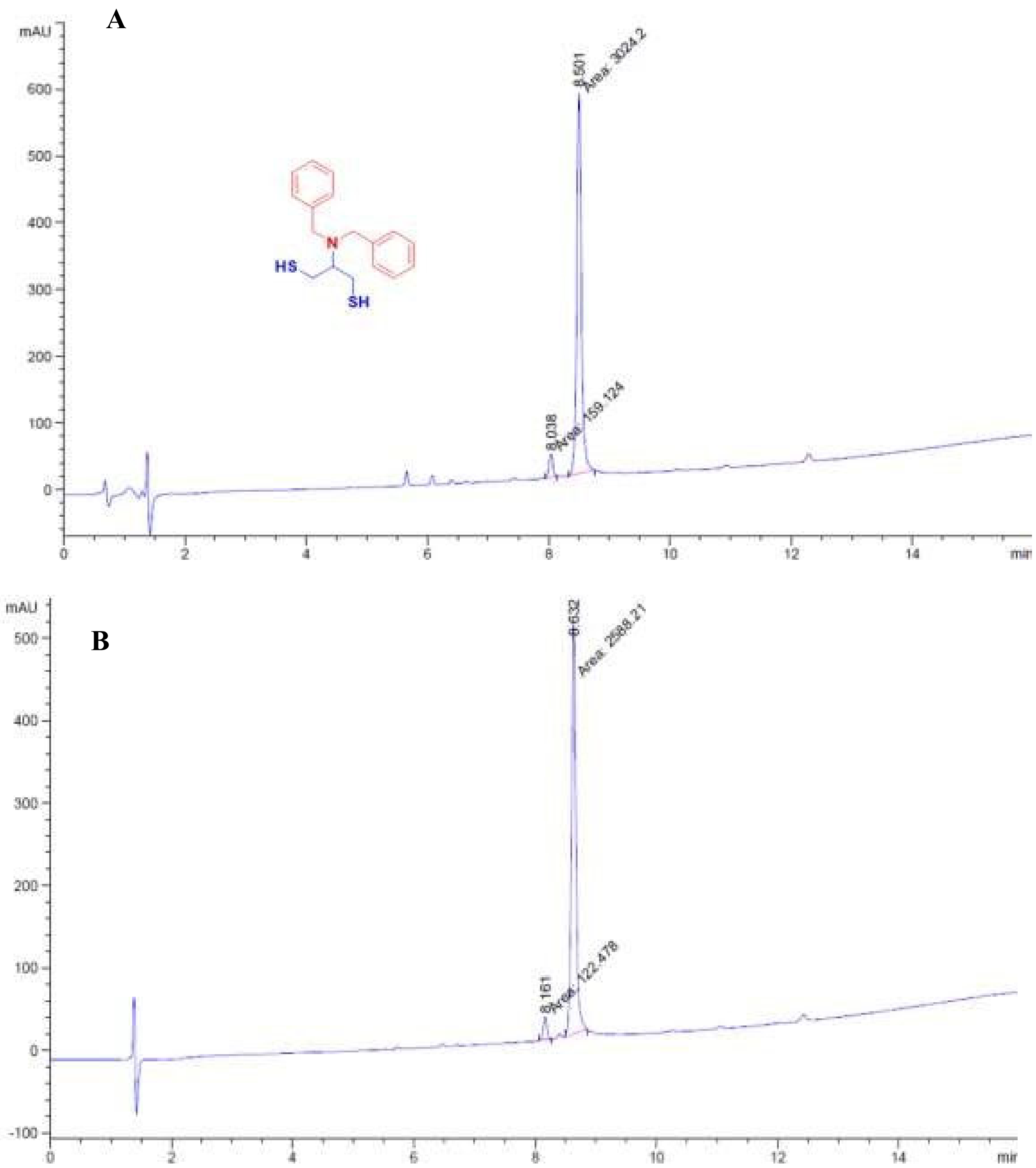


**Figure S33:** Stability test of DPDT in solid form. **(A)** freshly prepared; **(B)** after 60 days.

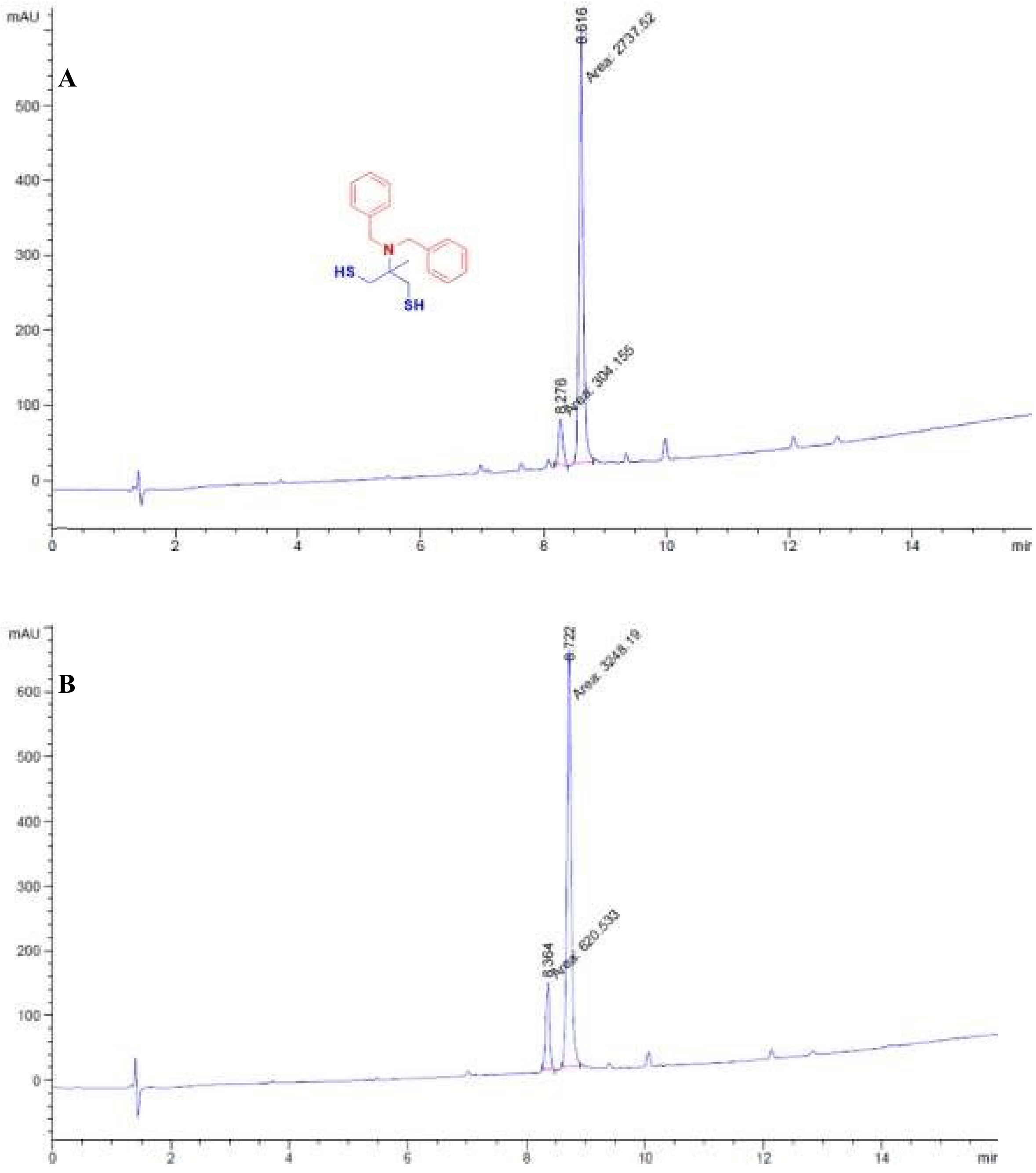


**Figure S34:** Stability test of DMPDT in solid form. **(A**) freshly prepared; **(B)** after 60 days.

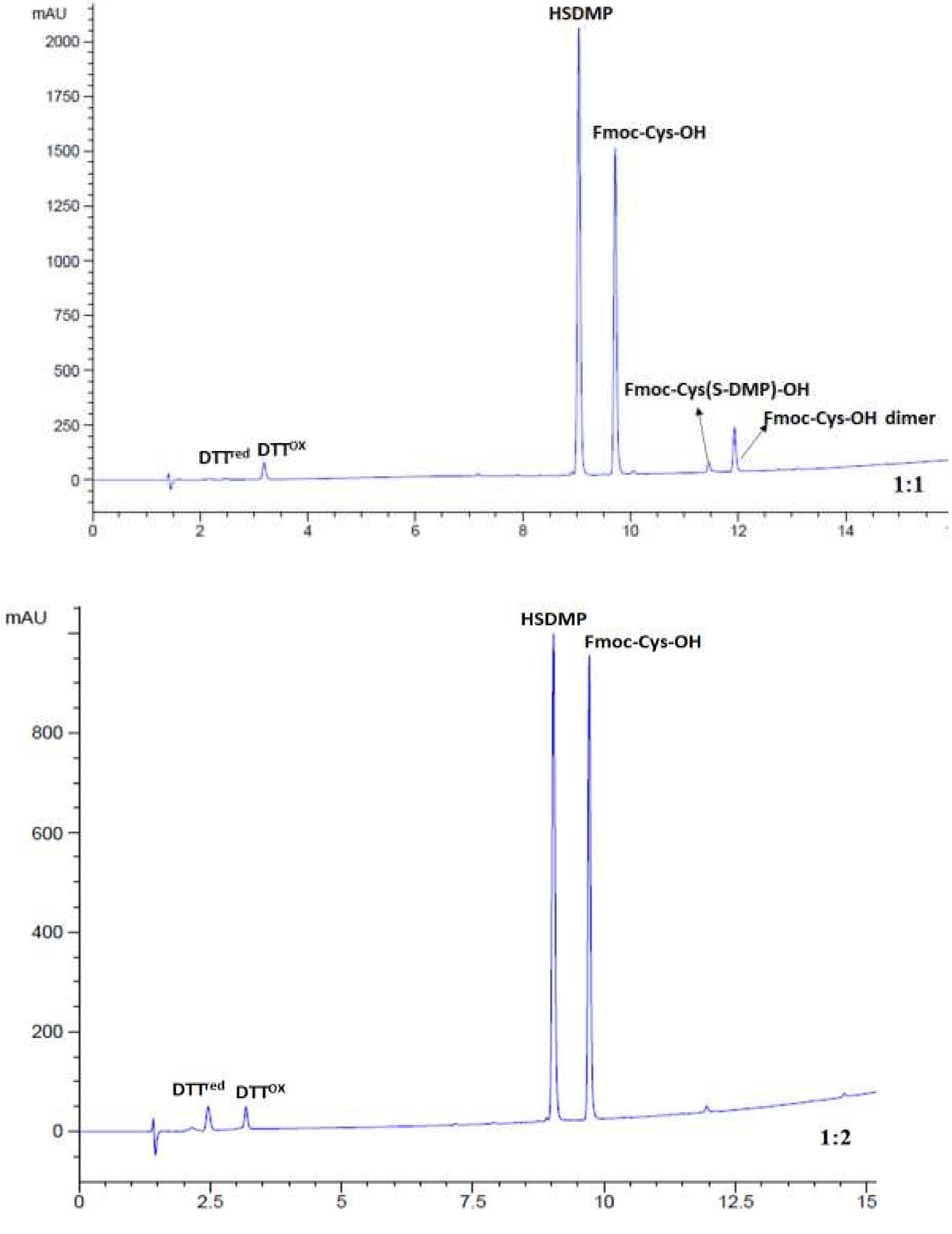


**Figure S35:** HPLC chromatogram of Fmoc-Cys(SDMP)-OH with DTT (1:1 and 1:2 ratio) with 2.5% DIEA and 2.5% water at 0 min.

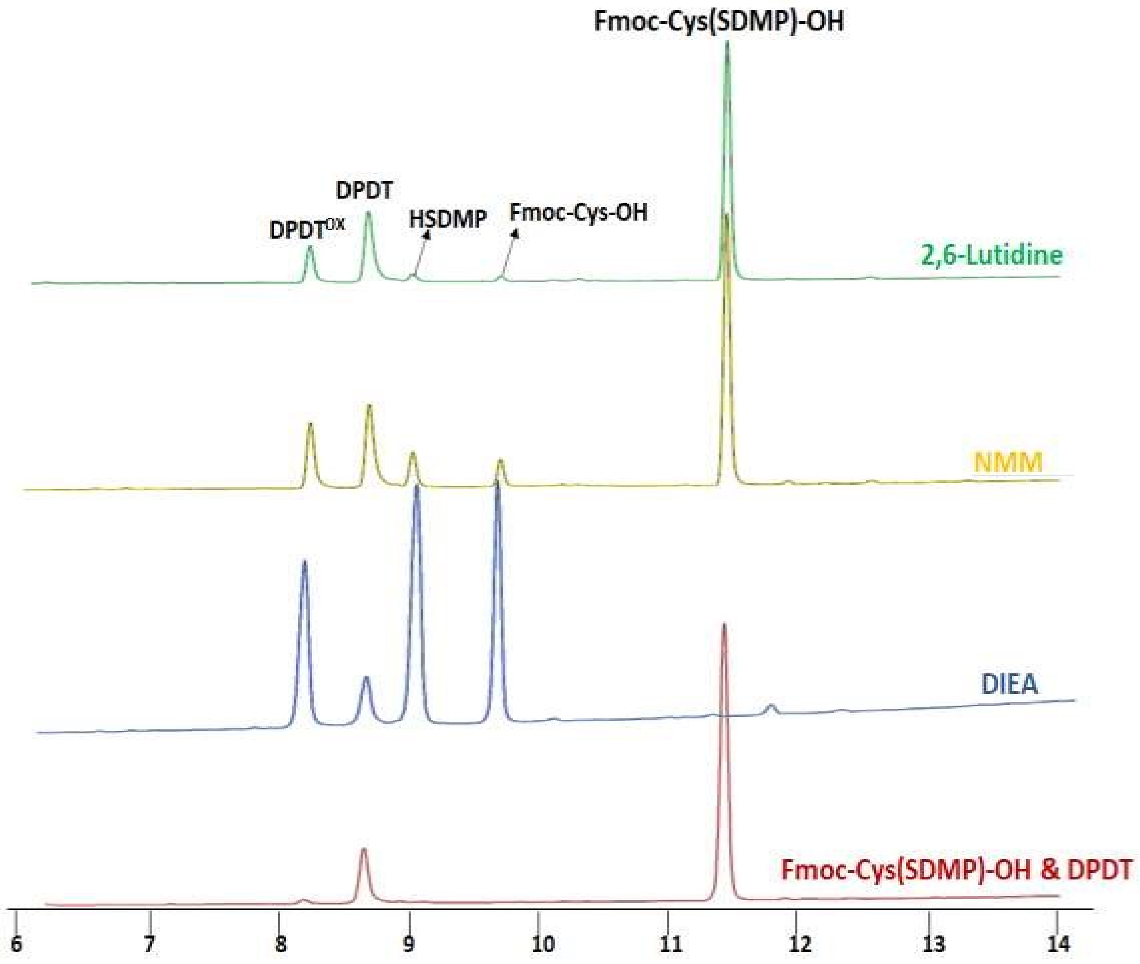


**Figure S36:** Effect of different bases in reduction of Fmoc-Cys(S-DMP)-OH using DPDT in ACN/base/$H_2O$ (95:2.5:2.5).

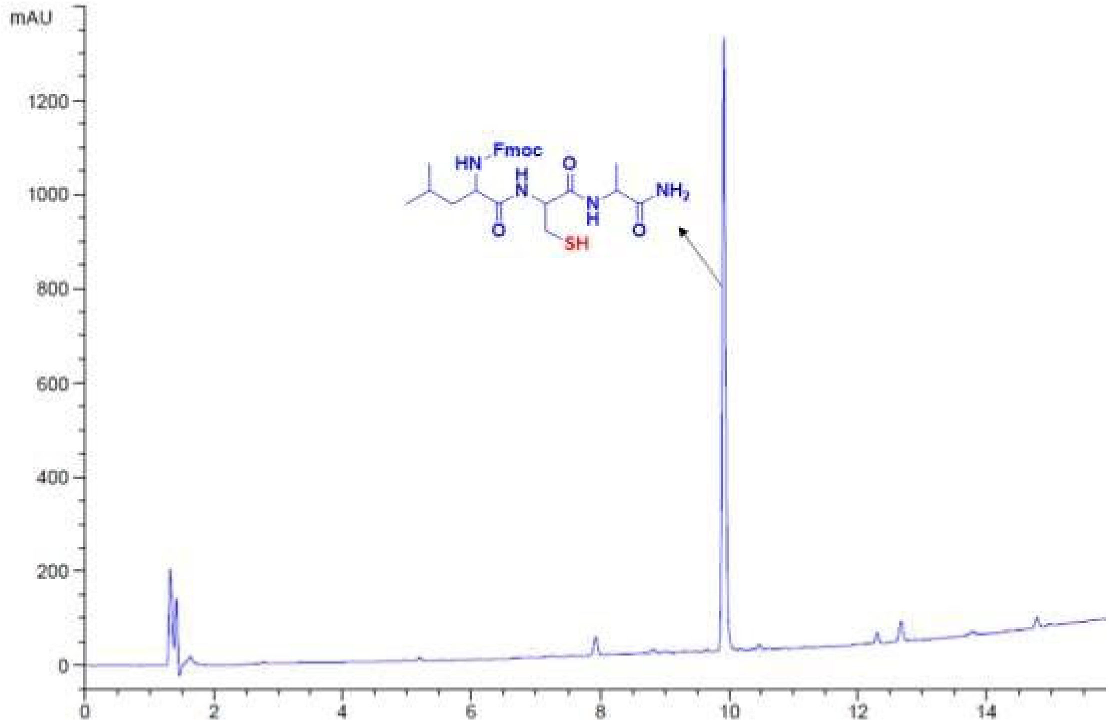


**Figure S37:** Fmoc-Ala-Cys(SIT)-Leu-NH-resin after treatment with DTT with 2.5% DIEA and 2.5% water in DMF.
